# Ciliary biology intersects autism and congenital heart disease

**DOI:** 10.1242/dev.204295

**Published:** 2025-06-24

**Authors:** Nia Teerikorpi, Kate E. McCluskey, Ethel Bader, Micaela C. Lasser, Sheng Wang, Catherine H. Nguyen, James D. Schmidt, Elina Kostyanovskaya, Nawei Sun, Jeanselle Dea, Tomasz J. Nowakowski, A. Jeremy Willsey, Helen Rankin Willsey

**Affiliations:** ^1^ Department of Psychiatry and Behavioral Sciences, University of California, San Francisco, San Francisco, CA 94143, USA; ^2^ UCSF Weill Institute for Neurosciences, University of California San Francisco, San Francisco, CA 94158, USA; ^3^ Department of Neurological Surgery, University of California, San Francisco, San Francisco, CA 94158, USA; ^4^ Department of Anatomy, University of California, San Francisco, San Francisco, CA 94158, USA; ^5^ Eli and Edythe Broad Center for Regeneration Medicine and Stem Cell Research, University of California, San Francisco, San Francisco, CA 94158, USA; ^6^ Chan Zuckerberg Biohub – San Francisco, San Francisco, CA 94158, USA

**Keywords:** Autism spectrum disorder, Congenital heart disease, Cilia, Tubulin, Proliferation, CRISPRi

## Abstract

Autism spectrum disorder (ASD) and congenital heart disease (CHD) frequently co-occur, yet the underlying molecular mechanisms of this comorbidity remain unknown. Given that children with CHD are identified as newborns, understanding which CHD variants are associated with autism could help select individuals for early intervention. Autism gene perturbations commonly dysregulate neural progenitor cell (NPC) biology, so we hypothesized that CHD genes disrupting neurogenesis are more likely to increase ASD risk. Therefore, we performed an *in vitro* pooled CRISPR interference screen to identify CHD genes disrupting NPC biology and identified 45 CHD genes. A cluster of ASD and CHD genes are enriched for ciliary biology, and perturbing any one of seven such genes (*CEP290*, *CHD4*, *KMT2E*, *NSD1*, *OFD1*, *RFX3* and *TAOK1*) impairs primary cilia formation *in vitro*. *In vivo* investigation of *TAOK1* in *Xenopus tropicalis* reveals a role in motile cilia formation and heart development, supporting its prediction as a CHD gene. Together, our findings highlight a set of CHD genes that may carry risk for ASD and underscore the role of cilia in shared ASD and CHD biology.

## Teaser

**Highlighted Article:** The increased likelihood of autism in individuals with congenital heart disease may stem from shared genetic mechanisms centered on cilia biology.

## INTRODUCTION

Autism spectrum disorders (ASD) are complex neurodevelopmental conditions that commonly co-occur with congenital heart disease (CHD) (Marino et al., 2012; Bean Jaworski et al., 2017; Gu et al., 2023). Both ASD and CHD are highly heritable and share genetic determinants (Zaidi et al., 2013; De Rubeis et al., 2014; Homsy et al., 2015; Jin et al., 2017; Willsey et al., 2018a; Satterstrom et al., 2020). Using joint network propagation of ASD and CHD genes, we previously identified significant overlap of associated molecular networks, and pinpointed chromatin modification, NOTCH signaling, MAPK signaling and ion transport as potential areas of shared biology (Rosenthal et al., 2021). Together, there is strong evidence that ASD and CHD likely share common biology, yet the underlying molecular mechanisms remain unclear.

Because CHD is generally identified before birth, the common co-occurrence with ASD affords an opportunity for early identification of individuals who may develop ASD, conduct observational studies and begin interventions sooner than typically possible (Homsy et al., 2015; Jin et al., 2017; Willsey et al., 2018a). This strategy will be most effective if individuals with CHD can be stratified by their likelihood of developing ASD, yet we cannot currently do this due to the relatively limited overlap between high-confidence genes identified in rare variant-based whole-exome sequencing studies of ASD and of CHD (Jin et al., 2017; Satterstrom et al., 2020). Therefore, we have employed a multiplexed *in vitro* genetic screen to prioritize CHD genes likely to increase the odds of ASD.

*In vivo* and *in vitro* studies have repeatedly identified neural progenitor cell (NPC) proliferation as a convergent phenotype in ASD (Packer, 2016; Marchetto et al., 2017; Sacco et al, 2018; Courchesne et al., 2019; Iakoucheva et al., 2019; Lalli et al., 2020; Willsey et al., 2021, 2022; Sun et al., 2024 preprint). We hypothesized that the subset of CHD genes that disrupt neurogenesis may increase the likelihood of ASD. Therefore, we performed a multiplexed CRISPR interference (CRISPRi) proliferation and survival screen (Tian et al., 2019) in human NPCs, targeting ASD and CHD genes. Overall, we identified 45 CHD genes that strongly impact NPCs, as well as a cluster of ASD-CHD genes that impact NPCs and are putatively involved in ciliary biology. Within this cluster, we showed that all seven genes predicted to contribute to ASD and CHD (*CEP290*, *CHD4*, *KMT2E*, *NSD1*, *OFD1*, *RFX3* and *TAOK1*) impact primary cilia development in human cells. We also demonstrated that loss of *TAOK1* impairs motile cilia, as well as heart and brain development in *Xenopus*. These results not only identify a set of CHD genes that are likely associated with an increased ASD likelihood but also suggest that cilia play a significant role in the shared biological mechanisms of both disorders, consistent with recent findings linking tubulin and ciliary biology to ASD (Lasser et al., 2023; Sun et al., 2024 preprint; Kostyanovskaya et al., 2025 preprint).

## RESULTS

### Pooled proliferation and/or survival screen of ASD and CHD genes in NPCs

**Fig. 1.**
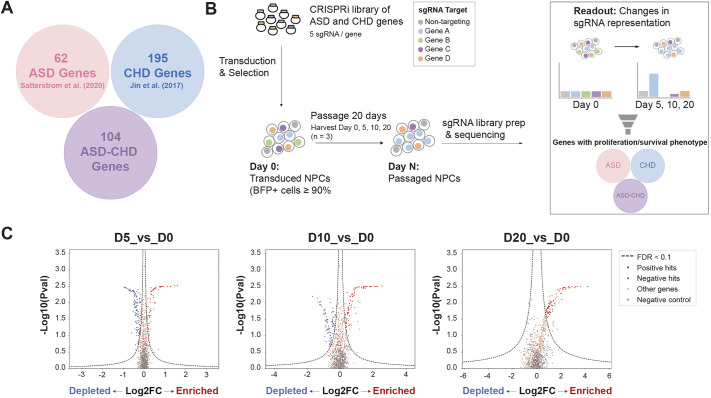
**Pooled proliferation and/or survival screen of autism spectrum disorder and congenital heart disease genes in neural progenitor cells.** (A) Schematic showing autism spectrum disorder (ASD) genes (pink; according to Satterstrom et al., 2020), congenital heart disease (CHD) genes (blue; according to Jin et al., 2017) and ASD-CHD genes (purple; 21 share genetic risk, according to Satterstrom et al., 2020 and Jin et al., 2017). Sixty eight have predicted shared risk (Rosenthal et al., 2021) and/or 34 are CHD genes (according to Jin et al., 2017) that are present in the SFARI database. (B) Strategy for CRISPRi screen. Neural progenitor cells (NPCs) are harvested at days 0, 5, 10 and 20, and sgRNA representation at day 5, 10 and 20 is compared against day 0. (C) Volcano plots summarizing knockdown phenotypes and statistical significance (Mann–Whitney U test). Dashed lines indicate cutoff for hit sgRNAs (FDR=0.1), highlighting sgRNAs that are significantly enriched (red circles) or significantly depleted (blue circles). Gray circles represent non-targeting sgRNAs and orange circles represent non-significant genes. See also Table S1.

ASD gene variants commonly perturb NPCs (Packer, 2016; Marchetto et al., 2017; Sacco et al., 2018; Courchesne et al., 2019; Iakoucheva et al., 2019; Willsey et al., 2021, 2022; Sun et al., 2024 preprint). To investigate whether CHD gene variants similarly affect NPCs, we leveraged a bulk CRISPRi screening approach (Tian et al., 2019). We generated a pooled lentiviral sgRNA library targeting 62 ASD genes, 195 CHD genes and 104 ‘ASD-CHD’ shared genes, using at least five sgRNAs per gene (361 total genes, Fig. 1A, see Materials and Methods for gene selection criteria) and 255 non-targeting control sgRNAs (Tian et al., 2019). Next, we generated NPCs from the Allen Institute for Cell Science (AICS) dCAS9 iPSC line, which enables stable CRISPRi in iPSC-derived neuronal lines (Tian et al., 2019; Sun et al., 2024 preprint). These NPCs were transduced with the sgRNA library and passaged for 20 days. We collected cells at days 0, 5, 10 and 20, and the abundance of each sgRNA was determined by sequencing sgRNA protospacers (Fig. 1B). We used MAGeCK (Li et al., 2014; Tian et al., 2019) to compare sgRNA representation at each timepoint versus day 0 and calculated gene-level fold-changes and false discovery rates (FDRs) as described by Tian et al. (2019). Overall, we identified 24 ASD, 77 CHD and 44 ASD-CHD genes that impact survival and/or proliferation of NPCs when disrupted (FDR<0.1, 145 total genes, Fig. 1C, Table S1), supporting our hypothesis that a subset of CHD genes disrupt neurogenesis.

### Enrichment of ciliary biology among ASD and CHD genes

**Fig. 2.**
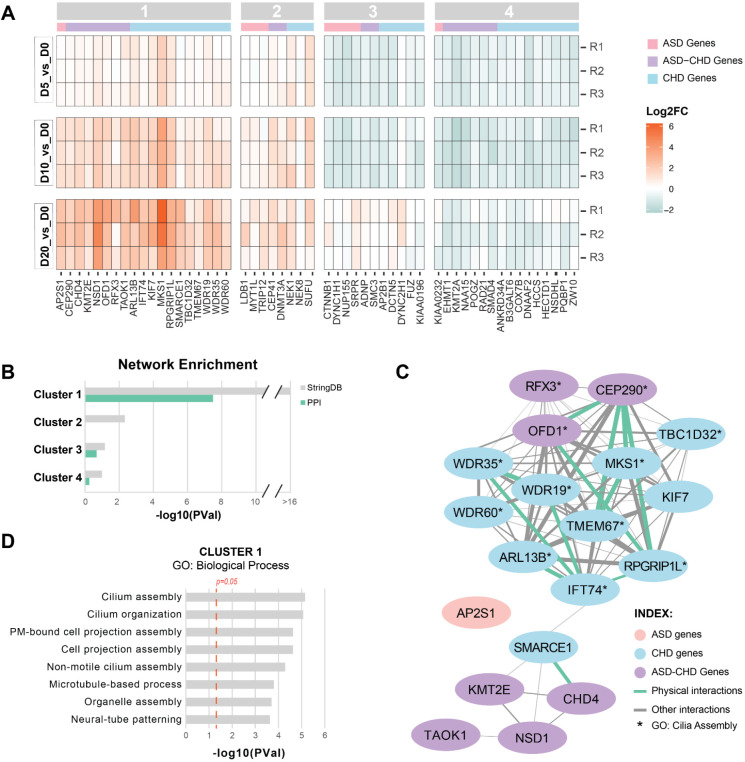
**A subset of ASD and CHD genes converges on cilia biology.** (A) Heat-map showing gene knockdowns clustered by k-means (see Fig. S1). R1-R3 represent biological replicates. Cutoff for genes is *P*<0.05 and absolute value of log2(fold-change)≥0.585 for at least one timepoint. ASD genes are represented by pink bars, CHD genes are represented by blue bars and ASD-CHD genes are represented by purple bars. (B) Genes within cluster 1 are more connected than expected by chance in StringDB. Enrichment is shown for all interactions in StringDB (gray) or for only physical interactions (green). *P*-values are not corrected for multiple comparisons. (C) Cluster 1 gene StringDB network. ASD genes (pink), CHD genes (blue), ASD-CHD genes (purple), physical interactions (green) or other StringDB interaction (gray) are shown. (D) Cluster 1 genes are enriched for biological process GO terms related to cilia (ToppGene using a background of the 361 genes screened; red line, *P*=0.05). Only terms with FDR<0.05 (Benjamini-Hochberg) are displayed. See Tables S2 and S3.

To subset the 145 significant genes into groups that potentially represent convergent biological processes, we focused on the 54 genes (9 ASD, 28 CHD and 17 ASD-CHD) with a fold-change in guide representation>1.5× at one or more timepoints (absolute log2FC≥0.585, *P* value≤0.05, Table S2) and performed k-means clustering (Fig. 2A). While there are two obvious clusters (enriched versus depleted sgRNAs), we created four clusters based on elbow plot and silhouette methods (Fig. S1A,B). All clusters contained ASD, CHD and ASD-CHD genes. To identify clusters with more gene connections than expected by chance, which would suggest shared biology, we queried StringDB, a database of known and predicted physical and functional interactions (Szklarczyk et al., 2021). This analysis identified Cluster 1 as containing the only gene set with a significant enrichment of interactions in the full StringDB and protein-protein interaction networks (Fig. 2B,C, Fig. S2).

To identify potential biological pathways or processes, we used ToppGene (Chen et al., 2009) to perform gene ontology (GO) enrichment analysis of the cluster 1 genes, using the 361 genes screened here as background. Eight ‘Biological Process’ terms were significantly enriched (Benjamini-Hochberg FDR<0.05, *P* value≤0.05, Fig. 2D), with the top seven terms related to cilia, which are microtubule-based organelles essential for proliferation, patterning, signaling and survival (Anvarian et al., 2019; Zaidi et al., 2022; Mill et al., 2023). The eighth term was ‘Neural-tube patterning’, a process that is reliant on cilia (Guemez-Gamboa et al., 2014). Similarly, all eight significantly enriched ‘Cellular Component’ terms relate to cilia and microtubule biology (Table S3). These results suggest that genes in cluster 1, when perturbed, could impact cilia, leading to changes in both brain and heart development (Guemez-Gamboa et al., 2014; Youn and Han, 2018; Djenoune et al., 2022; Shaikh Qureshi and Hentges, 2024).

### ASD-CHD shared genes impact ciliary biology

Based on the enrichment of cluster 1 genes for ciliary GO terms, we directly tested whether individual disruption of a subset results in cilia defects. We selected the seven ASD-CHD genes (*CEP290*, *CHD4*, *KMT2E*, *NSD1*, *OFD1*, *RFX3* and *TAOK1*), as these genes are most likely to represent shared biology. Of these genes, four (*CEP290*, *CHD4*, *OFD1* and *RFX3*) have previously described roles in cilia (Marley and von Zastrow, 2012; Rachel et al., 2015; Chen et al., 2018; Firat-Karalar, 2018; Robson et al., 2019; Wu et al., 2020; Morleo et al., 2023). The remaining three (*KMT2E*, *NSD1* and *TAOK1*) have not been directly implicated in ciliary biology. However, *KMT2E* and *TAOK1* regulate microtubules (Draviam et al., 2007; Zhao et al., 2016), which are the main cytoskeletal component of cilia.

**Fig. 3.**
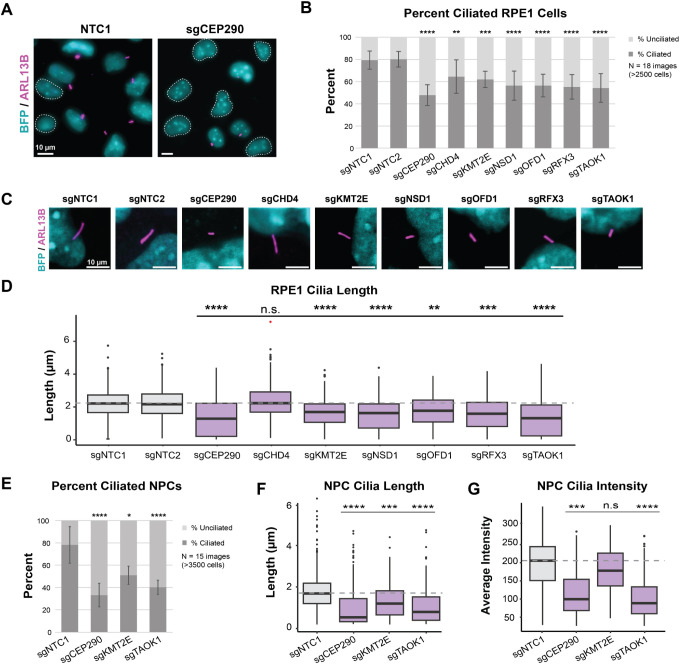
**Knockdown of ASD-CHD genes disrupts primary cilia.** (A) Representative image of ciliated RPE1 cells transduced (BFP^+^) with non-targeting control sgRNA (sgNTC1) or *CEP290* sgRNA (sgCEP290). BFP^+^ cells (cyan) without primary cilia (ARL13B, magenta) are outlined with a white dotted line. (B) ASD-CHD gene (*CEP290*, *CHD4*, *KMT2E*, *NSD1*, *OFD1*, *RFX3* and *TAOK1*) repression decreases the percentage of ciliated cells (≥2500 cells: 18 images, three biological replicates each). Data are mean±s.e.m. (C) Representative image of cilia length in RPE1 cells for gene knockdowns. (D) Primary cilia length decreases for some ASD-CHD gene knockdowns (≥250 cells: 15 images, three biological replicates each). (E) Percentage of cilia phenotypes replicated in neural progenitor cells (NPCs) (≥3500 cells across 15 images, three biological replicates each). Data are mean±s.e.m. (F) Cilia length phenotypes replicated in NPCs (≥350 cells across 15 images, three biological replicates each). (G) We also observed a decrease in ARL13B signal for *CEP290* and *TAOK1* knockdowns. All data are normalized based on average cell density of the non-targeting control sgRNA. Data before normalization are in Fig. S4. In the box and whisker plots, boxes indicate the IQR, the midline is the median, the upper whiskers indicate the upper quartile+1.5 (IQR), the lower whiskers indicate the lower quartile-1.5 (IQR) and the asterisks represent outliers. Significance (Dunn's multiple comparisons): *P<0.05; **P<0.01; ***P<0.001; ****P<0.0001; n.s., not significant (*P>*0.05). See also Figs S3 and S4.

To determine the extent to which these ASD-CHD genes play a role in ciliary biology, we individually disrupted expression using CRISPRi in immortalized mitotically arrested retinal pigment epithelial cells (RPE1), which provides a robust *in vitro* model for evaluating primary cilia (May-Simera et al., 2018). All seven genes are expressed in these cells, and we confirmed strong knockdown by qPCR (Fig. S3A). We measured the percentage of ciliated cells and cilia length (Fig. S3B,C), and normalized these data based on average blue fluorescent protein (BFP)^+^ cell density of the non-targeting control sgRNA 1 (Fig. 3, Fig. S4). Repression of each of these seven ASD-CHD genes resulted in a significant decreases in the percentage of ciliated cells when compared to two independent, non-targeting controls (Fig. 3A,B, Figs S3B, S4A). Additionally, except for *CHD4*, repression of each gene resulted in decreased cilia length (Fig. 3C-D, Figs S3C, S4B). Together, these data provide evidence that these seven ASD-CHD genes intersect ciliary biology.

Next, to assess whether cilia function during brain development may be compromised, we selected three of these genes (*CEP290*, *KMT2E* and *TAOK1*) for experiments in human iPSC-derived NPCs. These genes represent different degrees of evidence for ciliary relevance and ASD/CHD association. *CEP290* is a well-characterized CHD gene (Jin et al., 2017) present in the SFARI list of ASD genes (categories 1-2, S) with a known role in cilia as a component of the basal body (Firat-Karalar, 2018). In contrast, *TAOK1* and *KMT2E* are ASD genes (Satterstrom et al., 2020; Fu et al., 2022) with predicted risk for CHD based on network propagation (Rosenthal et al., 2021), but they have no known role at the cilium. As in RPE1 cells, we assessed the percentage of ciliated cells and cilia length, and observed significant alterations of both after individual knockdown (Fig. 3E,F, Fig. S4C,D). Within cilia, we observed a decrease in ARL13B intensity for *CEP290* and *TAOK1* knockdowns (Fig. 3G, Fig. S4E), suggesting that these genes may impact ARL13B expression and/or ciliary localization. *TAOK1* knockdown does not significantly change cell cycle phase progression in NPCs (Fig. S5A-C), so, at least for this gene, ciliation defects do not seem to be secondary to cell cycle issues. Together, these results demonstrate that these predicted ASD-CHD genes impact cilia biology.

### *TAOK1* depletion disrupts brain and heart development *in vivo*

**Fig. 4.**
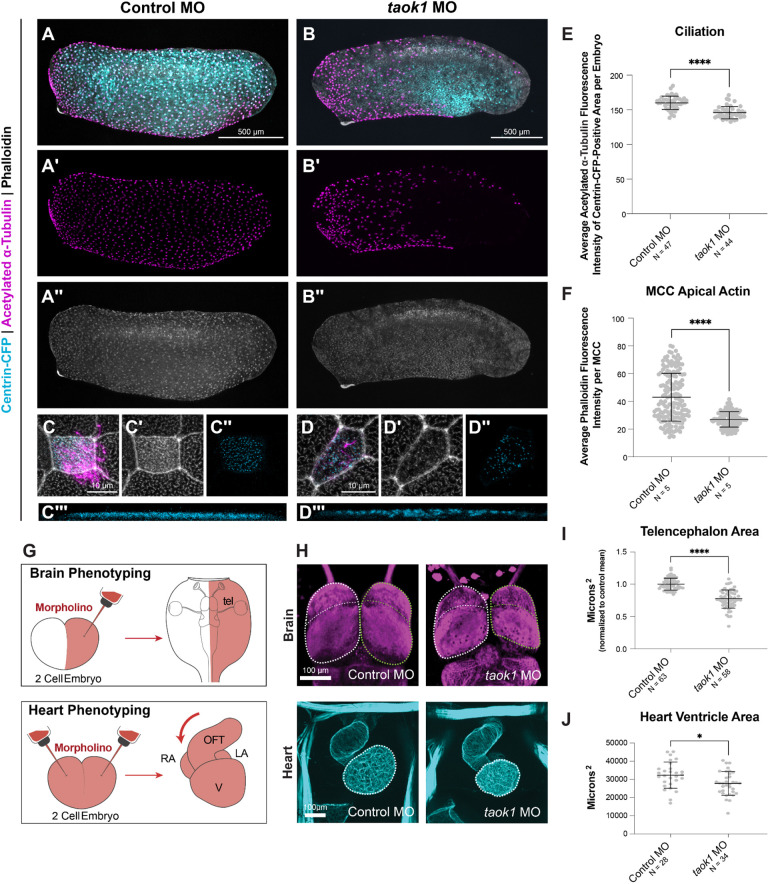
***TAOK1* disruption perturbs ciliation, and brain and heart development *in vivo*.** (A-B″) Depletion of *taok1* by a translation-blocking morpholino (MO) reduces ciliation compared to control morpholino-injected NF stage 28 *X. tropicalis* stained for acetylated a-tubulin (cilia, magenta) and with phalloidin (actin, gray), and injected with Centrin-CFP mRNA (basal bodies, blue). Scale bars: 500 µm. (C-D‴) *taok1* depletion also reduces phalloidin and disrupts basal body distribution. Scale bars: 10 µm. (E) Acetylated ɑ-tubulin quantification within centrin-positive areas per embryo (*taok1* MO, *n*=44; control MO, *n*=47). (F) Quantification of apical actin (*taok1* MO, *n*=5 embryos, 124 cells; control MO, *n*=5 embryos, 161 cells). (G) Morpholino and a tracer (dextran) are injected at the two-cell stage into one cell (brain phenotyping) or into both cells (heart phenotyping). Telencephalon, tel; outflow tract, oft; ventricle, V; right atrium, RA; left atrium, LA). (H) *taok1* depletion decreases telencephalon size (top, β-tubulin, magenta) and heart ventricle size (bottom, phalloidin/actin, cyan). Scale bars: 100 µm. (I) Telencephalon size relative to the uninjected side (µm^2^). (J) Variation in heart ventricle size. ****P<0.0001 by Mann–Whitney rank sum test; *P<0.05 by non-parametric Mann–Whitney rank sum test. Data are mean±s.e.m. See Figs S6 and 7, and Movie 1.

While *TAOK1* is a high-confidence ASD gene (Satterstrom et al., 2020; Fu et al., 2022), its association with CHD has only been predicted by network propagation (Rosenthal et al., 2021). Therefore, we sought to elaborate its role *in vivo* in heart development and at motile cilia, which are implicated in CHD (Klena et al., 2017; Gabriel et al., 2021). To do this, we used the *Xenopus* epidermis as a model for motile cilia, due to their experimental tractability, and established CHD relevance (Duncan and Khokha, 2016; Garfinkel and Khokha, 2017; Kulkarni et al., 2018). We observed localization of TAOK1 at ciliary basal bodies and axonemes in motile multiciliated cells (MCCs) by both GFP-tagged overexpression of the human protein (Fig. S6A,B) and by endogenous antibody staining against Taok1 (Fig. S6C). Depletion of *taok1* in *Xenopus tropicalis* by translation-blocking morpholino caused a reduction in Taok1 antibody signal in MCCs (Fig. S6D-F) and a loss of ciliation in the epidermis (Fig. 4A-D), as measured by the average acetylated alpha-tubulin fluorescence intensity (*P*<0.0001) (Fig. 4A-E). Additionally, *taok1* depletion caused defects in basal body docking (Fig. 4C,D) and apical actin organization (Fig. 4C,D,F). We orthogonally perturbed *taok1* by CRISPR/Cas9 mutagenesis. Compared to control CRISPR (*slc45a2*), *taok1* CRISPR caused a loss of ciliation and apical actin, similar to the morpholino (Fig. S7). Together, our results demonstrate that TAOK1 is crucial for primary cilia *in vitro* and for motile cilia *in vivo*.

Next, we tested whether disruption of *taok1* leads to brain and heart phenotypes *in vivo*. For the brain, we created unilaterally *taok1*-depleted tadpoles by morpholino injection into one cell of two-cell stage *X. tropicalis* embryos (Fig. 4G) and observed significantly decreased telencephalon size (Fig. 4H,I). For hearts, we created bilaterally depleted embryos by morpholino injection into both blastomeres at the two-cell stage (Fig. 4G), and observed a decrease in heart ventricle size (Fig. 4H,J, Movie 1). These results suggest that *TAOK1* is required for brain and heart development, reinforcing its predicted role in ciliary biology hypothesized here, as cilia defects underlie many CHDs (Fakhro et al., 2011; Yuan et al., 2013; Li et al., 2015; Klena et al., 2016; Jin et al., 2017; Klena et al., 2017; Gabriel et al., 2021; Djenoune et al., 2022). Together, our work demonstrates that *TAOK1* impacts primary and motile cilia, heart development, and brain development.

## DISCUSSION

We have identified ASD, CHD and predicted ASD-CHD genes that impact NPC proliferation and/or survival, showing both *in vitro* and *in vivo* evidence implicating cilia biology at the intersection of ASD and CHD genetics. Specifically, we identified 24 ASD, 77 CHD and 44 ASD-CHD genes that perturb NPCs. Among these genes, we observed an enrichment of GO terms related to ciliary biology, highlighting cilia as a key organelle underlying ASD-CHD co-occurrence. We validated this finding, showing that repression of seven ASD-CHD predicted genes individually (*CEP290*, *CHD4*, *KMT2E*, *NSD1*, *OFD1*, *RFX3* and *TAOK1*) led to primary cilia defects *in vitro* in human cells. Finally, we identified an additional role for *TAOK1* in motile cilia as well as heart and brain development *in vivo* in *Xenopus*, supporting its predicted risk for CHD. This work provides the first direct evidence that ciliary biology underlies the comorbidity between ASD and CHD, establishing a molecular basis for this overlap and highlighting cilia as a previously unreported point of convergence for shared risk genes.

Regarding the mechanism by which TAOK1 affects ciliogenesis, we show that TAOK1 localizes to cilia and that disruption results in defects in ciliogenesis in *Xenopus*. These findings align with work showing that TAOK1 affects cytoskeletal stability (Byeon and Yadav, 2024) and other work identifying TAOK1 as a predicted regulator of TTBK2, which is a ciliary regulator (Loukil et al., 2021; Bashore et al., 2023). Additionally, we observed changes to ARL13B intensity in several of the *in vitro* conditions. Future work should explore whether each perturbation causes changes in ciliary trafficking and/or structure, and how these changes affect signaling and fluid flow.

Building on earlier studies identifying enrichment in chromatin regulation, NOTCH signaling and MAPK signaling for genes with shared risk for ASD and CHD (Fakhro et al., 2011; Zaidi et al., 2013; Rosenthal et al., 2021; Zaidi et al., 2022), our study adds ciliary biology as a point of vulnerability intersecting these disorders. Ciliary underpinnings in CHD are well established (Fakhro et al., 2011; Yuan et al., 2013; Li et al., 2015; Klena et al., 2016; Jin et al., 2017; Klena et al., 2017; Gabriel et al., 2021; Djenoune et al., 2022), but their implications for ASD are less appreciated despite emerging evidence (Marley and von Zastrow, 2012; Willsey et al., 2018b, 2020; Rosengren et al., 2018; Di Nardo et al., 2020; Frasca et al., 2020; Kostyanovskaya et al., 2025 preprint). This work, combined with our group's recent work showing that ASD-associated chromatin regulators also regulate microtubules (Lasser et al., 2023), sheds light on the shared ASD-CHD biology around chromatin regulation. Specifically, our findings suggest that enrichment of chromatin regulation genes may reflect a broader role at microtubules, as microtubules are the major structural component of cilia. Additional work from our group shows broader convergence of ASD genes onto microtubule biology (Sun et al., 2024 preprint), suggesting this ciliary finding may apply to broader ASD mechanisms beyond CHD comorbidity.

Cilia have diverse cellular functions, including regulating proliferation, differentiation and excitability (Malicki and Johnson, 2017; Mitchison and Valente, 2017; Tereshko et al., 2021; Mill et al., 2023). Conversely, changes to proliferation and differentiation can affect cilia formation (Kasahara and Inagaki, 2021), which makes it difficult to determine what was primarily affected in our screen. As our screen cannot disentangle impacts on proliferation, differentiation or survival, it is difficult to determine which process(es) is the cause of the shared phenotype. Further, the screen likely misses genes that would show phenotypes during differentiation, but not during proliferation. While many ASD gene variants cause NPC proliferation defects, it also remains unclear how the observed NPC phenotypes relate to ASD risk directly. General defects in microtubule stability will affect cell proliferation and/or survival via the mitotic spindle and cilia formation and length, so it is unclear which of these processes are central to the phenotypes observed here (and in patients). Nevertheless, we showed that these perturbations caused alterations in cilia length in mitotically arrested cells, supporting a direct role at cilia. Our work does not provide evidence that disruption of NPC proliferation is specific to ASD and/or CHD. Additionally, future work could use haploinsufficiency models to elaborate dose-dependent mechanisms. Cilia are also the sole site of hedgehog signaling (Bangs and Anderson, 2017), so the effect of ASD and/or CHD gene perturbation on differentiation and signaling during development is an exciting future direction. Finally, cilia have cell type-specific functions, so future work could explore how these genes affect cilia formation and function across cell types. Overall, our work provides insights into the shared biology underlying ASD and CHD, identifies a class of genes potentially associated with both conditions, and offers a foundation for exploring how these genes influence heart and brain development.

## MATERIALS AND METHODS

### Human cell culture

#### Human iPSCs

The Allen Institute for Cell Science (AICS) BFP-tagged dCas9-KRAB WTC iPSC line (AICS-0090-391, MONO-ALLELIC TagBFP-TAGGED dCas9-KRAB WTC) was cultured in mTESR Plus Medium (Stem Cell Technologies, 05825) on Matrigel (Fisher Scientific, 08-774-552)-coated cell culture dishes (Corning, 08-774-552) diluted in DMEM F12 (Fisher Scientific, 11320-082). mTESR Plus Medium was replaced every day and cells (70-90% confluent) were passaged using Accutase (Stem Cell Technologies, 07920), then re-plated in mTESR Plus Medium with the addition of 10 nM Y-27632 dihydrochloride ROCK inhibitor (Tocris, 125410) for 24 h.

#### Human iPSC-derived neural progenitor cells

We generated neural progenitor cells (NPCs) from the AICs dCAS9 iPSC line using a modified version of a monolayer dual-SMAD inhibition protocol combined with small molecules, producing >98% PAX6^+^ cells (Sun et al., 2024 preprint). Briefly, we treated cells with LDN193189, SB431542 and XAV939 for 6 days. The cells were then passaged and cultured with XAV939 alone for two more days to generate NPCs. NPCs were then maintained in N2/B27 medium (DMEM F-12, 1× B27 -Vit.A, 1× N2, 1× GlutaMAX, 1× MEM-NEAA, 10 ng/ml EGF and 10 ng/ml FGF2). The medium was changed every other day and cells were passaged at ∼90% confluence using Accutase.

#### Human RPE1/LentiX-293T

Immortalized hTERT dCas9 RPE1 (Jost et al., 2017) cells were cultured in DMEM F12 (ThermoFisher Scientific, 11320-082) supplemented with 10% FBS on Corning cell culture dishes. LentiX-293T cells were cultured in DMEM (Fisher Scientific, 10-566-024) supplemented with 1× MEM-NEAA and 10% FBS. Both RPE1s and 293Ts were passaged using 0.25% trypsin-EDTA. All cell lines were subjected to mycoplasma testing every 6 months. No contaminations were observed.

### Pooled proliferation/survival screen

Guides were designed using the CRISPRiaDesign tool (https://github.com/mhorlbeck/CRISPRiaDesign; Horlbeck et al., 2016). We selected 100 high-confidence ASD-risk (Satterstrom et al., 2020) and 248 CHD-risk (Jin et al., 2017) genes from studies that have leveraged the statistical power of recurrent rare *de novo* variants in ASD probands. We also identified 104 shared risk ‘ASD-CHD’ genes defined by having at least one of the following characteristics: (1) being present in both ASD and CHD gene lists (Jin et al., 2017; Satterstrom et al., 2020), (2) being CHD genes (Jin et al., 2017) found in the SFARI Gene Database (Gene score: 1-2, Syndromic) or (3) predicted to share risk by network proximity analysis (Rosenthal et al., 2021). Due to overlap between these three gene sets (ASD, CHD and ASD-CHD), we ultimately designed a library targeting 361 total genes (62 high-confidence ASD genes, 195 CHD genes and 104 ‘ASD-CHD’ shared risk genes). We designed five sgRNAs per gene and selected 255 non-targeting control sgRNAs (10% total sgRNA). Guides were cloned into pMK1334 [CROPseq-Guide-Puro vector (Tian et al., 2019), RRID:Addgene_ 127965; gifted by Martin Kampmann]. To select non-targeting control sgRNAs for this experiment, we used a set of 225 sequences designed by the Kampmann Lab to have significant mismatches to any known genomic target (Tian et al., 2021). We then screened for correctly assembled clones by colony PCR and further validated them using Sanger sequencing. The library balance of sgRNA sequences were then assessed and verified by Ion Torrent Sequencing.

To produce lentivirus for our CRISPRi library, we used LentiX-293T (Clontech) that were maintained in DMEM with Glutamax (Fisher Scientific,10566016), MEM-NEAA (Fisher Scientific, 11140-050) and 10% FBS. Lentiviral packaging was performed by seeding 2.4 million cells per 10 cm dish, then transfecting with 2.5 µg equimolar packaging mix (pMDL, pRSV and pVSV-g), 2.5 µg sgRNa vector (PMK1334) using OptiMEM and Lipofectamine 2000 (Fisher Scientific, 11668019). 72 h later, we collected the supernatant, filtered with a 0.45 µm PVDF syringe filter and concentrated the virus using the Lenti-X Concentrator (Takara Bio, 631231). HEK293 media was replaced with DMEM-F12 when concentrating the virus.

The concentrated virus containing the validated sgRNA library was transduced into NPCs through pooled packaging at 10-20% efficiency to ensure one integration event per cell. We seeded 3 million NPCs each onto two matrigel-coated 10 cm dishes and added 100 µl of concentrated virus that had been resuspended in 1 ml of DMEM F12. Two days later, cells were passaged. One million cells were taken for FACS sorting on BFP to ensure no more than 20% transduction efficiency, while the rest were re-plated onto three matrigel-coated 10 cm dishes (three replicates/3 million cells per plate) in N2/B27 media containing 3 µg/ml puromycin (Fisher Scientific, 501532829) to select for the pMK1334 sgRNA vector. We refreshed selection media daily, then on day 3 we passaged cells using accutase after washing three times to remove dead cells. Approximately 1 million cells were fluorescence activated cell sorted (FACS) to ensure ≥85% of cells from each of the three replicate conditions were expressing BFP. Then 5 million cells from each replicate (≥85% BFP^+^) were harvested (day 0; library representation ∼1000 cells per sgRNA). The remainder of the cells were re-plated onto three 10 cm cell culture dishes for later time points. We seeded 2 million NPCs per plate and cultured in N2/B27 medium, as described previously (Sun et al., 2024). The cells were passaged every 3-5 days and approximately 5 million cells from each replicate were then harvested at days 5, 10 and 20, after validating ≥85% BFP^+^ cells via FACS. We isolated genomic DNA from all samples using the Zymo Quick DNA mini-prep Plus Kit (D4068). The samples were amplified and prepared for sequencing as described previously (Gilbert et al., 2014).

### Pooled proliferation and/or survival screen – data analysis

Data were analyzed using a bioinformatics pipeline, MAGeCK-iNC (MAGeCK including Negative Controls) as previously described (Li et al., 2014; Tian et al., 2019). Briefly, to determine sgRNA counts in each sample, we cropped and aligned the raw sequencing reads to the reference using Bowtie (Li et al., 2014). Next, we removed outlier data points (sgRNA count coefficient of variation ≥1). Count's files of timepoints to be compared were then input into MAGeCK to generate log2 fold changes (Log2FC) and *P*-values for each sgRNA, using the ‘mageck tesk -k’ command. We subtracted the median Log2FC of non-targeting sgRNA from gene-targeting sgRNA to assess changes in gene-targeting sgRNA representation at each timepoint. Gene-level knockdown effects were then determined by taking the mean of individual sgRNA scores for the top 3 sgRNAs targeting a specific gene. Screen-positive genes were selected based on a gene-level false discovery rate (FDR) of less than 0.1.

We then further prioritized genes with a *P*-value of less than 0.05 and an absolute gene-level Log2FC greater than or equal to 0.585 for at least one time-point for k-means clustering via Morpheus (https://software.broadinstitute.org/morpheus), estimating an ideal cluster number of four using the elbow plot method and silhouette method (Fig. S1). We performed gene ontology enrichment analysis of the Cluster 1 genes with ToppGene (J. Chen et al., 2009), using the 361 genes from our CRISPRi library as background. We considered gene ontology terms with Benjamini-Hochberg FDR<0.05 as significantly enriched and calculated fold-enrichment using the following formula: (hit count in input genes/hit count in background)/(number of input genes/number of background genes).

### CRISPRi imaging screen

The sgRNAs with the strongest phenotype from the pooled proliferation and/or survival screen were selected to generate individual sgRNA KD cell lines for CEP290, CHD4, KMT2E, NSD1, OFD1, RFX3 and TAOK1, as well as two non-targeting controls. CRISPRi cell lines were generated as previously described (Willsey et al., 2021; Sun et al., 2024 preprint), from hTERT dCas9 RPE1s and AICS dCas9 iPSC-derived NPCs. Knock-down was confirmed by qPCR using the ΔΔCT method (Fig. S3A). RPE1 cells were plated on a 96-well glass bottom plate (Corning, CLS3603) at a density of 2×10^4^ cells per well. NPCs were plated on a matrigel-coated 96-well glass bottom plate at 4×10^4^ cells per well. RPE1s were serum starved (DMEM-F12 -FBS), then both RPE1s and NPCs were fixed after 24 h in 4% paraformaldehyde. We permeabilized cells for 15 min in PBST (PBS and 0.2% Triton X-100) and blocked in blocking buffer (PBS, 0.2% Triton X-100 and 2% BSA) for 45 min at room temperature. Cells were incubated in blocking buffer with primary antibody overnight at 4°C. ARL13B primary antibody (1:500, ProteinTech, 17711-1-AP) was used to visualize cilia. The cells were then washed three times in PBST for a total of 45 min and incubated for 1 h at room temperature in a blocking buffer with goat anti-rabbit secondary antibody (1:1000, Fisher Scientific, A32732) as well as DRAQ5 (1:500, Fisher Scientific, 5016967). Stained cells were then washed three times in PBST for a total of 45 min, before being stored at 4°C in PBS for imaging. Images were acquired using a Zeiss 980 LSM confocal microscope with 20× and 63× objectives.

### CRISPRi imaging screen – data analysis

Cilia count was determined using the CellProfiler 4.2.5 software (McQuin et al., 2018). We adapted the ‘Speckle Counting’ pipeline to reliably identify cilia. First, the ARL13B channel was enhanced to remove background noise. BFP^+^ nuclei (positively transduced cells) were identified using a diameter range of 40-140 pixel units, threshold range of 0.0-1.0, threshold strategy set to ‘Global’ and threshold method set to ‘Minimum Cross-Entropy’. Cilia of these BFP^+^ cells were counted using a diameter range of 5-30 pixel units, a threshold range of 0.2-1.0, threshold strategy set to ‘Global’ and threshold method set to ‘Otsu’. We determined the percentage of ciliated cells in each image by dividing the number of cilia identified within a given image by the total number of cells present in the same image (Fig. S4). To normalize this measure, we divided the percentage of ciliated cells by the ratio of the number of cells in the image to the average BFP^+^ cell density of the non-targeting control sgRNA (Fig. 3A,B,E). Statistical significance was determined using Dunn's multiple comparisons test in Graphpad (Prism).

Cilia length was determined using the CiliaQ plug-in on Fiji (Hansen et al., 2021). Briefly, cilia length was quantified by inputting *z*-stacks of ARL13B and BFP channels into ‘CiliaQ Preparator’. Images were checked by eye for errors in cilia identification and colocalization with BFP^+^ cells. Errors in cilia identification were corrected using the ‘CiliaQ Editor’. Finally, cilia intensity and length were calculated using ‘CiliaQ V0.1.4’ with minimum cilium size (voxel) set to 20. Cilia length measurements were again normalized based on average BFP^+^ cell density of the non-targeting control sgRNA, and cilia intensity measurements were normalized based on average DRAQ5 fluorescence. Statistical significance was determined using Dunn's multiple comparisons test in Graphpad (Prism). We only quantified positively transduced BFP^+^ cells for all CRISPRi assays.

### *Xenopus* husbandry and microinjection

Male and female wild-type *Xenopus laevis* and *Xenopus tropicalis* were maintained and cared for according to established IACUC protocols. Ovulation was induced in females using human chorionic gonadotropin (Sigma) according to Sive et al (2000) before performing natural matings or *in vitro* fertilizations. Localization work was carried out in *X. laevis,* while knockdown was carried out in *X. tropicalis*.

Human *TAOK1* cDNA sequence (NM_020791.4) was cloned into the GFP vector (C-terminal tag) pcDNA3.1+ and injected at 20 pg per blastomere at the four-cell stage, targeting the epidermis in *Xenopus laevis*, with or without 100 pg of Centrin-CFP mRNA.

A Zeiss Stemi 508 microscope, Narishige micromanipulator and a Narishige IM-400 injector were used to inject reagents for *Xenopus* experiments. Animals were fixed and stained at NF stage 28 (Nieuwkoop, 1994) for epidermal imaging or NF stage 46 for heart and/or brain imaging. For morpholino experiments, we generated a translation-blocking *taok1* morpholino (MO) (5′-TTGTTGACGGCATCCTGCTTCAG-3′) to disrupt *taok1* expression in *X. tropicalis* or a standard control morpholino (5′-CCTCTTACCTCAGTTACAATTTATA-3′) purchased from Gene Tools. For cilia phenotype analysis, we injected 5.53 ng of *taok1* MO or standard control along with 100 pg of Centrin-CFP RNA per embryo into one cell at the four-cell stage. For heart and/or brain phenotyping, 3.32 ng of *taok1* MO or standard control along with a dextran tracer, was injected unilaterally at the two-cell stage for brain phenotyping, or in both cells at the two-cell stage for heart phenotyping.

For CRISPR experiments, we synthesized (EnGen, NEB E3322S) and purified (Zymo R1018) an sgRNA targeting *taok1* or a previously validated control sgRNA targeting *slc45a2* (Willsey et al., 2021). We designed our *taok1* sgRNA against the *X. tropicalis* genome version 10 with CRISPRscan algorithm (Moreno-Mateos et al., 2015) and it was predicted to have no off-targets (target sequence: 5′-GTAAGTCTGATGCAGATCCTAGG-3′ oligo sequence: 5′-ttctaatacgactcactataGTAAGTCTGATGCAGATCCTAGGgttttagagctaga-3′). We injected 800 pg of sgRNA and 4.48 ng of Cas9-NLS protein (UC Berkeley MacroLabs; Lingeman et al., 2017) into one cell of 4-cell stage *X. tropicalis* embryos, along with 100 pg Centrin-CFP mRNA.

### *Xenopus* immunofluorescence staining

Stage 28 *X. tropicalis* embryos were fixed using 4% PFA diluted in PBS. Immunostaining was performed according to Willsey et al. (2018b), without bleaching. For endogenous localization, Taok1 primary antibody (1:100, Fisher Scientific, MABC292) and acetylated ɑ-tubulin primary antibody (1:1000, Abcam ab179484) were used along with goat anti-mouse Alexa Fluor 488 (1:250, ThermoFisher A32723) and goat anti-rabbit Alexa Fluor 555 (1:250, ThermoFisher A32732) secondary antibodies. Phalloidin (1:400, LifeTech A22287) was added during secondary antibody incubation. For morpholino validation, Taok1 primary antibody was used along with goat anti-mouse Alexa Fluor 555 (1:250, ThermoFisher A32727) secondary antibody. For exogenous hTAOK1-GFP staining, acetylated ɑ-tubulin primary antibody (1:1000, Abcam ab179484) was used with goat anti-rabbit Alexa Fluor 555 (1:250, ThermoFisher A32732) secondary antibody and phalloidin (1:400, LifeTech A22287) added during secondary antibody incubation.

For cilia phenotyping, acetylated ɑ-tubulin primary antibody (1:3000, Sigma T6793) along with goat anti-mouse Alexa Fluor 647 (1:250, ThermoFisher A32728)-conjugated secondary antibody were used to visualize cilia. Phalloidin (1:400, LifeTech A34055) was added during secondary antibody incubation to visualize actin.

For heart and/or brain phenotyping, NF stage 46 tadpoles were fixed with 4% PFA in PBS and immunostaining was performed according to Willsey et al. (2018a,b), with the omission of the bleaching step whenever phalloidin was included. Acetylated ɑ-tubulin primary antibody (1:500, Sigma T6793) along with goat anti-mouse Alexa Fluor 488 (1:500, LifeTech A32723) conjugated secondary antibodies were used to visualize the brain. Phalloidin (1:500, LifeTech A22287) was used to visualize the heart (actin).

### *Xenopus tropicalis* cilia phenotyping

Samples were mounted on glass slides (within an area enclosed by a ring of vacuum grease) with PBS and coverslipped. For whole-embryo phenotyping, we imaged mounted samples on a Zeiss AxioZoom V16 with a 1× objective and then measured the average intensity of acetylated ɑ-tubulin within the centrin-positive area (for morpholino experiments) or on the entire injected side of the embryo (for CRISPR experiments) using a custom FIJI macro. For morpholino analysis, a two-tailed Mann–Whitney rank sum test was performed to compare measurements between conditions in Prism (GraphPad). For CRISPR analysis, after passing for normality, a two-tailed *t*-test was performed to compare measurements between control and *taok1* knockdown conditions in Prism. For basal body and actin measurements, the multiciliated epidermis was imaged on a Zeiss LSM980 confocal microscope with a 63× oil objective. Images were acquired as *z*-stacks at system-optimized intervals and processed in FIJI as maximum intensity projections. To assess actin intensity, centrin-positive cells were circled within their cell boundary and phalloidin average intensity was measured in FIJI. A two-tailed Mann–Whitney rank sum test was performed to compare measurements between control and *taok1* knockdown conditions in Prism.

### *Xenopus tropicalis* heart/brain phenotyping

Heart and brain phenotyping was performed according to Rosenthal et al. (2021). Animals were imaged on a Zeiss AxioZoom V16 with a 1× objective. Brain region size was calculated from stereoscope images of brain immunostainings using the freehand select and measure functions in Fiji. The injected side was compared to the uninjected side (internal control). These measurements were from two-dimensional images taken from a dorsal perspective and are a reflection of relative size differences, not a direct quantification of cell number. Heart ventricle size was measured using the freehand select and measure functions in Fiji. Quantitative differences in heart ventricle size were calculated by comparing mean surface area between control versus *taok1* MO-injected embryos. For both brain and heart phenotyping, statistical significance was determined using an unpaired Mann–Whitney rank sum tests in Prism (Graphpad). For timelapse imaging of heartbeating, NF stage 46 *X. tropicalis* tadpoles were anesthetized in 0.02% tricaine and positioned in agarose molds with their ventral side upwards. Animals were imaged on a Zeiss AxioZoom V16 with a 1× objective with 140 ms intervals for 30 s. Videos were trimmed to 30 frames each and processed in FIJI.

## Supplementary Material



10.1242/develop.204295_sup1 Supplementary information

Table S1. List of screened genes (Log2FC, PVal, ASD-satterstrom, CHD-Jin, ASD-CHD Rosenthal, ASD-CHD Genetic, CHD-SFARI).

Table S2. List of significant genes input for k-means clustering (per replicate, Cluster number, Log2FC, PVal, Category (ASD, CHD, ASD-CHD)). *Legend: Dx = day number; Rx = replicate number

Table S3. All significant (FDR < 0.05) ToppGene Enrichments (Biological Process and Cellular Component) of Cluster 1 genes with CRISPRi screen genes used as background correction.
