# Supplementary material for: Ciliary biology intersects autism and congenital heart disease
Source: Development. 2025 Jun 24;152(12):dev204295. doi: 10.1242/dev.204295 (PMC12273630; doi:10.1242/dev.204295)
Supplement: Supplementary information [file develop-152-204295-s1.pdf]

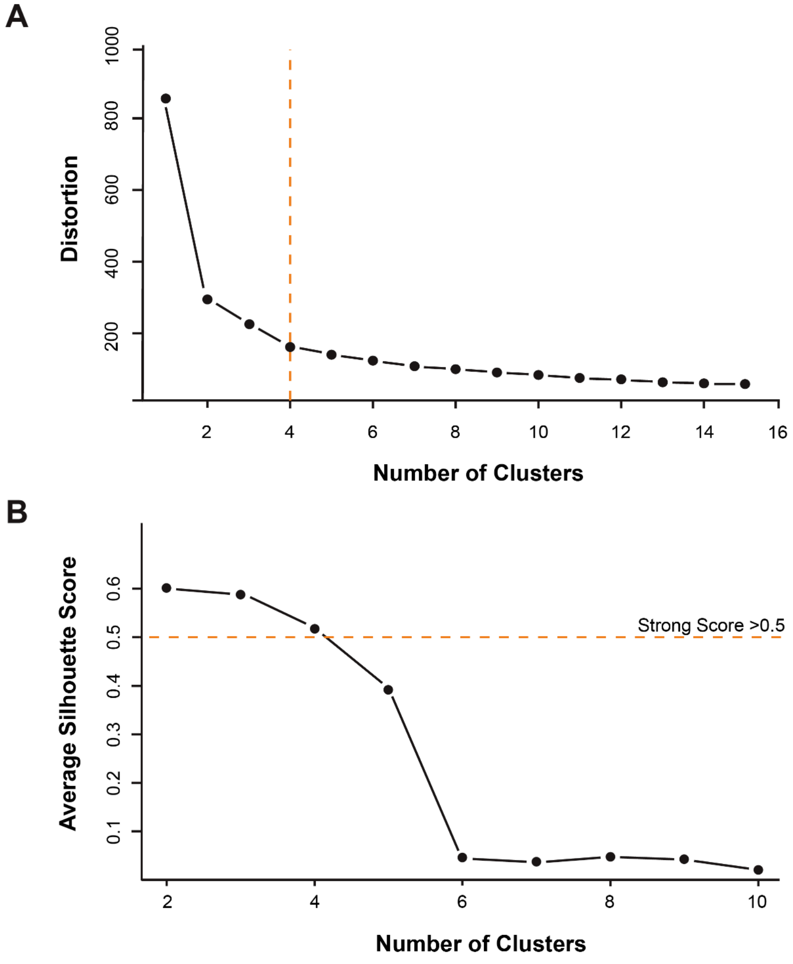

Fig. S1. Elbow Plot and Silhouette Method. (A) Elbow method was used to determine the optimal clustering number for our 54 proliferation/differentiation screen genes with FDR < 0.1 and |Log2FC| ≥ 0.585 in at least 1 timepoint. We determined four to be the optimal cluster number, by identifying the inflection point of the graph (orange dotted line). (B) Silhouette method was used to determine the optimal clustering number for our 54 proliferation/ differentiation screen genes with FDR < 0.1 and |Log2FC| ≥ 0.585 in at least 1 timepoint. A score above 0.5 (orange dotted line) indicates that the points are well-clustered, confirming that 4 is acceptable for k-means.

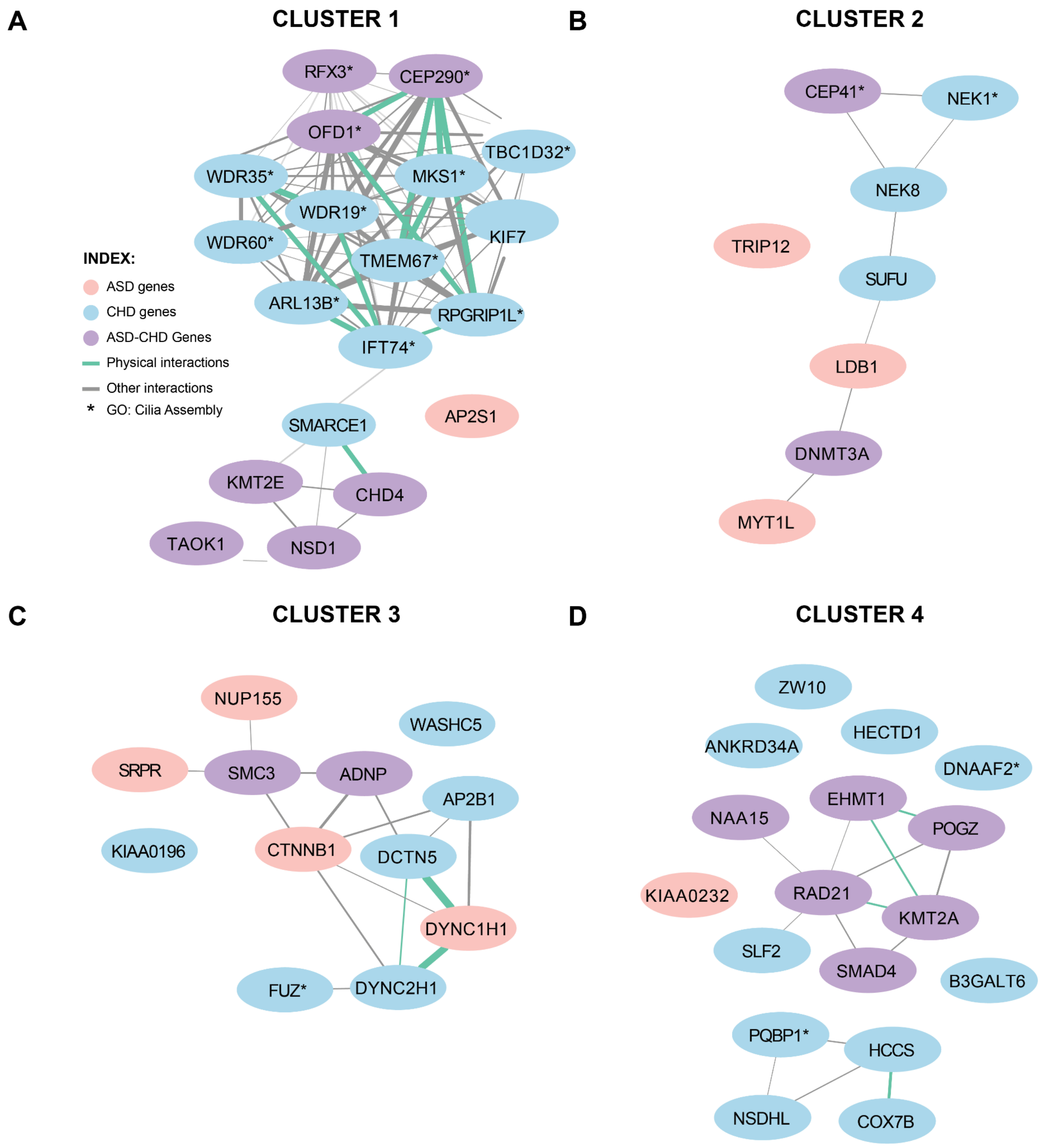

Fig. S2. Cluster 1 is significantly enriched for interactions. (A-D) Visualization of Cluster 1-4 interactions identified from StringDB. ASD-genes (Satterstrom, 2019) are represented by a pink circle, CHD-genes (Jin, 2017) are represented by a blue circle, and predicted ASD-CHD genes are represented by a purple circle. Physical interactions are connected with a green line and all other types of interactions are represented by grey.

Development • Supplementary information

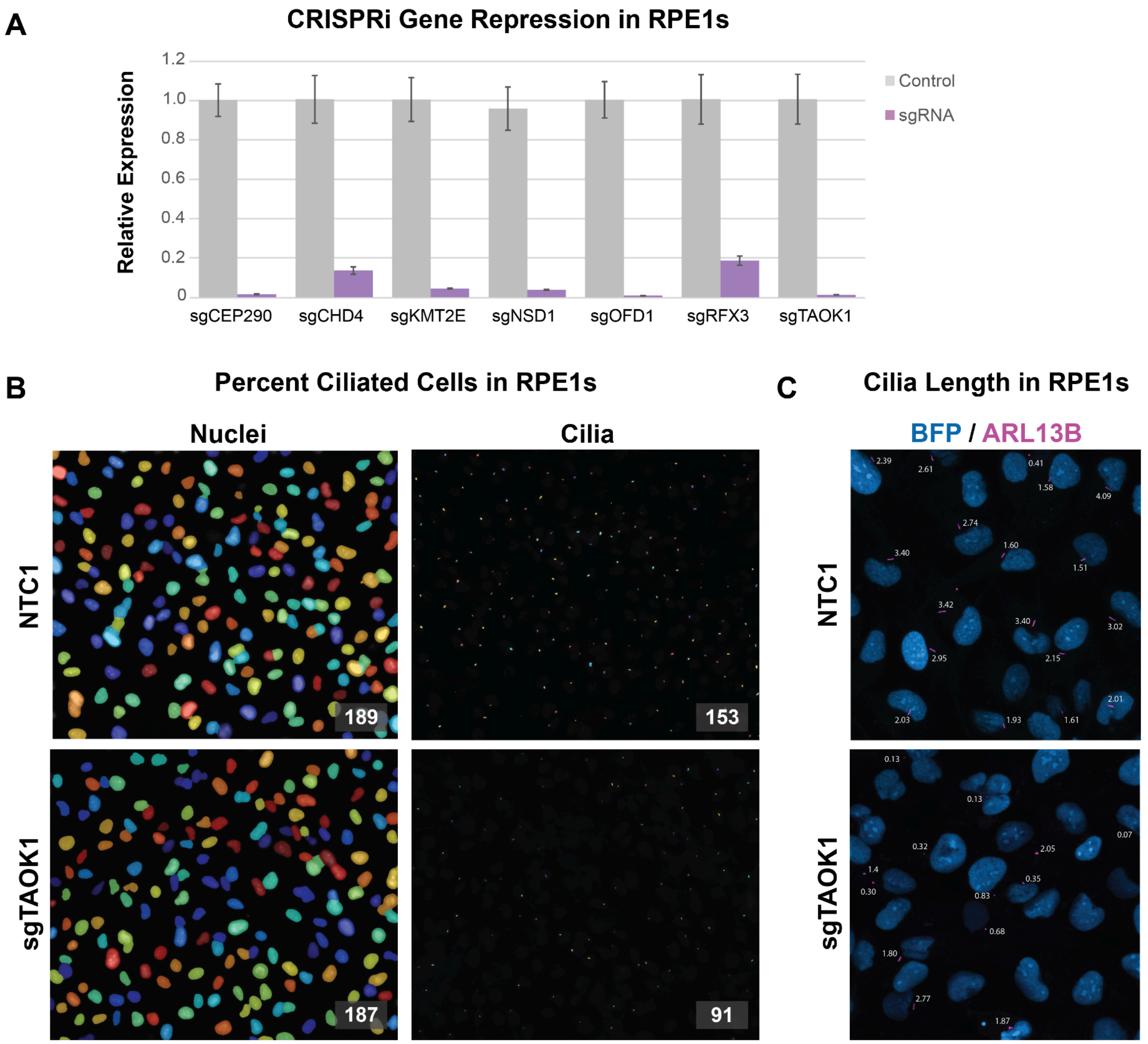

Fig. S3. Cilia quantification in RPE1 cells. (A) Knockdown efficiencies of the 7 ASD-CHD gene sgRNAs were evaluated individually in established RPE1 cell lines by qPCR. (B) Representative image of percent cilia quantification of non-targeting sgRNA (NTC1; 81% ciliated) and sgTAOK1 (49% ciliated) using CellProfiler. (C) Representative image of cilia length quantification of non-targeting sgRNA (NTC1; Average length: 2.38μm) and sgTAOK1 (Average length: 0.98μm) using CiliaQ. *Image is represented as a 2D maximum projection, while cilia length was measured in 3D.

Development • Supplementary information

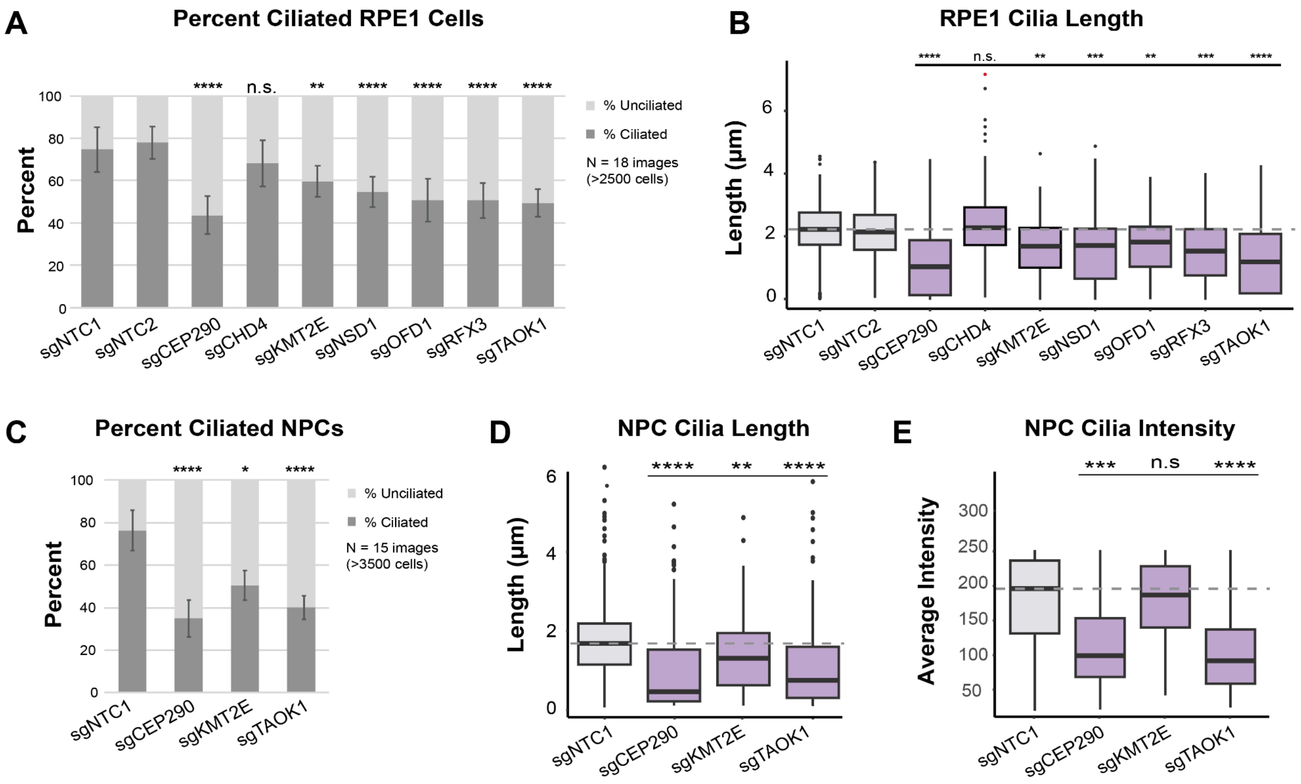

Fig. S4. Knock-down of ASD-CHD genes disrupts primary cilia (without normalization) (A) We quantified percent ciliated cells in ≥2500 RPE1 cells across 18 images (3 biological replicates). (B) We captured ≥250 cells across 15 images (3 biological replicates) for quantification of cilia length (µm) in RPE1 cells. (C) We captured ≥3500 cells across 15 images (3 biological replicates) for quantification of percent ciliated cells in NPCs. (D) We captured ≥350 cells across 15 images (3 biological replicates) in NPCs. (E) Using the images from (F), we measured ARL13B intensity (a.u.) for quantification of cilia intensity in NPCs.

**Significance (Dunn's multiple comparisons)*: *p < 0.05; ***p* < 0.01; ***p < 0.001; *****p* < 0.0001; n.s., not significant (p > 0.05).

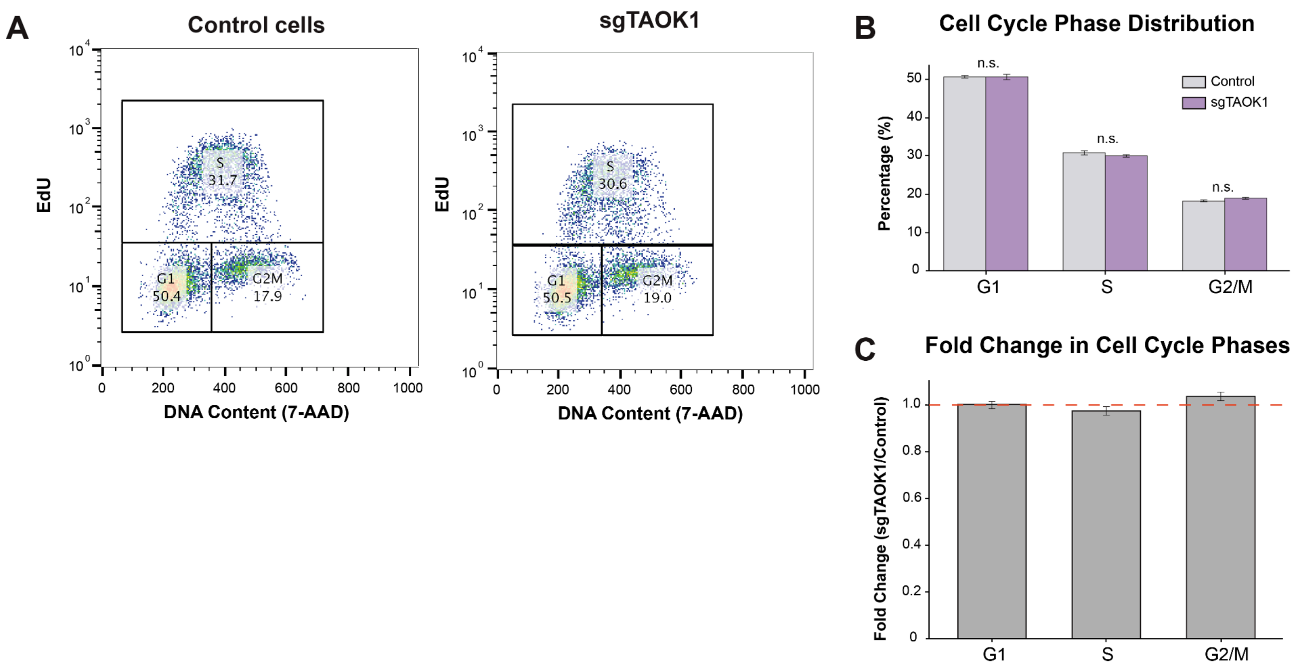

Fig. S5. Knock-down of *TAOK1* in NPCs does not significantly affect cell cycle phase occupancy. (A) Cell cycle phase occupancy measured by flow cytometry of NPCs carrying a non-targeting control sgRNA (Control) or *TAOK1* sgRNA (sgTAOK1). DNA content was labeled with 7-AAD and S phase cells were labeled with EdU. (B) Bar plot showing the percentage of cells in each cell cycle phase (G1, S, and G2/M) for control (grey, n=6) and sgTAOK1 (purple, n=3). Statistical analysis was performed using Mann-Whitney rank sum test. These results suggest that sgTAOK1 treatment does not significantly alter the distribution of cells at any phase of the cell cycle. (C) Bar plot showing the fold change in the percentage of cells in each cell cycle phase (G1, S, and G2/M) for sgTAOK1-treated cells relative to control. The dashed orange line at y = 1 indicates no change relative to control.

Development • Supplementary information

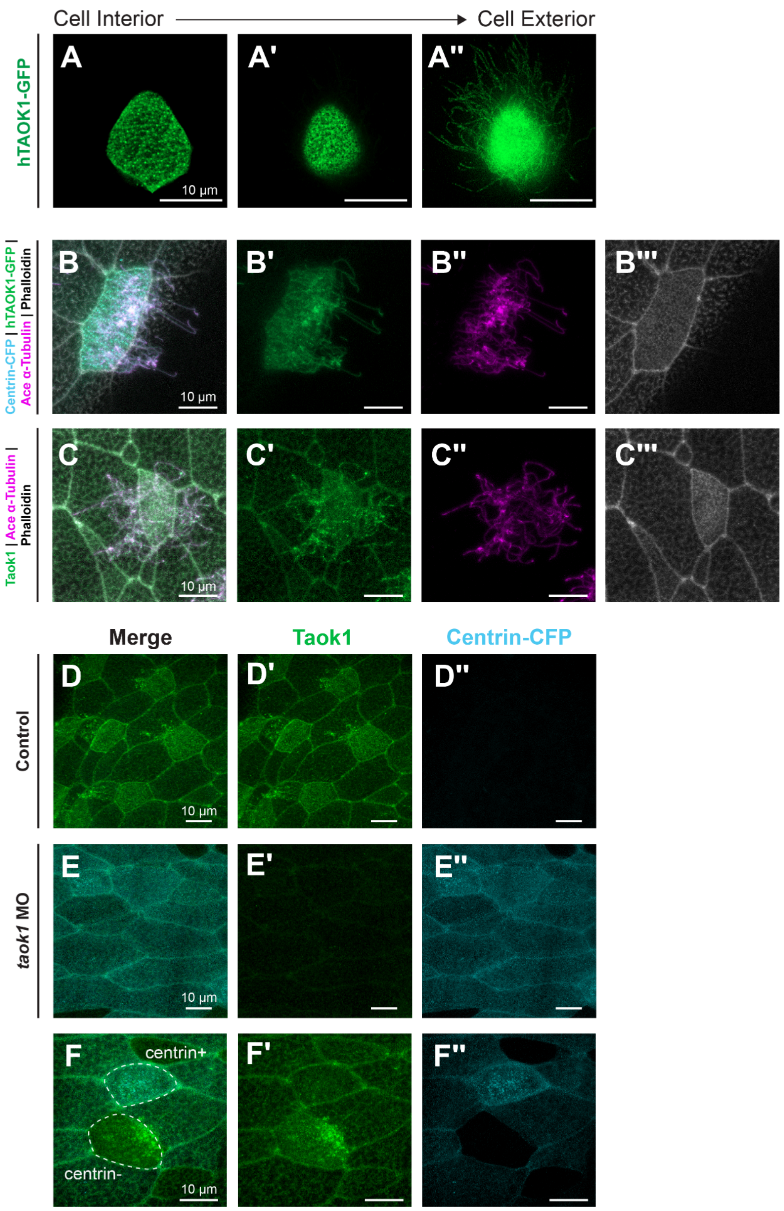

Fig. S6. TAOK1 localizes to ciliary structures (A - A''') hTAOK1-GFP injected into *X. laevis* localizes to ciliary structures of epidermal multiciliated cells. Images depict a single cell across several focal planes from cell interior to exterior. hTAOK1-GFP appears to localize to basal bodies (A), actin (A'), and ciliary axonemes (A''). These images are from an animal injected only with the GFP construct. (B - B''') Co-injection of hTAOK1-GFP with Centrin-CFP (Basal bodies, cyan), stained for acetylated alpha-tubulin (Cilia, magenta) and phalloidin (Actin, grey) to show overlap with GFP signal on cilia. (C - C''') Taok1 (green) antibody staining labels ciliary axonemes (Acetylated α-Tubulin, magenta) and the actin network (Phalloidin, gray) of the *X. tropicalis* epidermis. (D - F) Depletion of *taok1* reduces antibody staining of Taok1. (D - D'') Uninjected region of *X. tropicalis* epidermis staining (as shown by lack of centrin-positive cells) shows staining of endogenous Taok1 antibody. (E - E'') *X. tropicalis* epidermis co-injected with *taok1* morpholino and Centrin-CFP shows reduced antibody staining for Taok1. (F - F'') Co-injected multiciliated cell (Centrin-positive) shows reduced Taok1 staining compared to adjacent unmanipulated multiciliated cell (Centrin-negative).

Development • Supplementary information

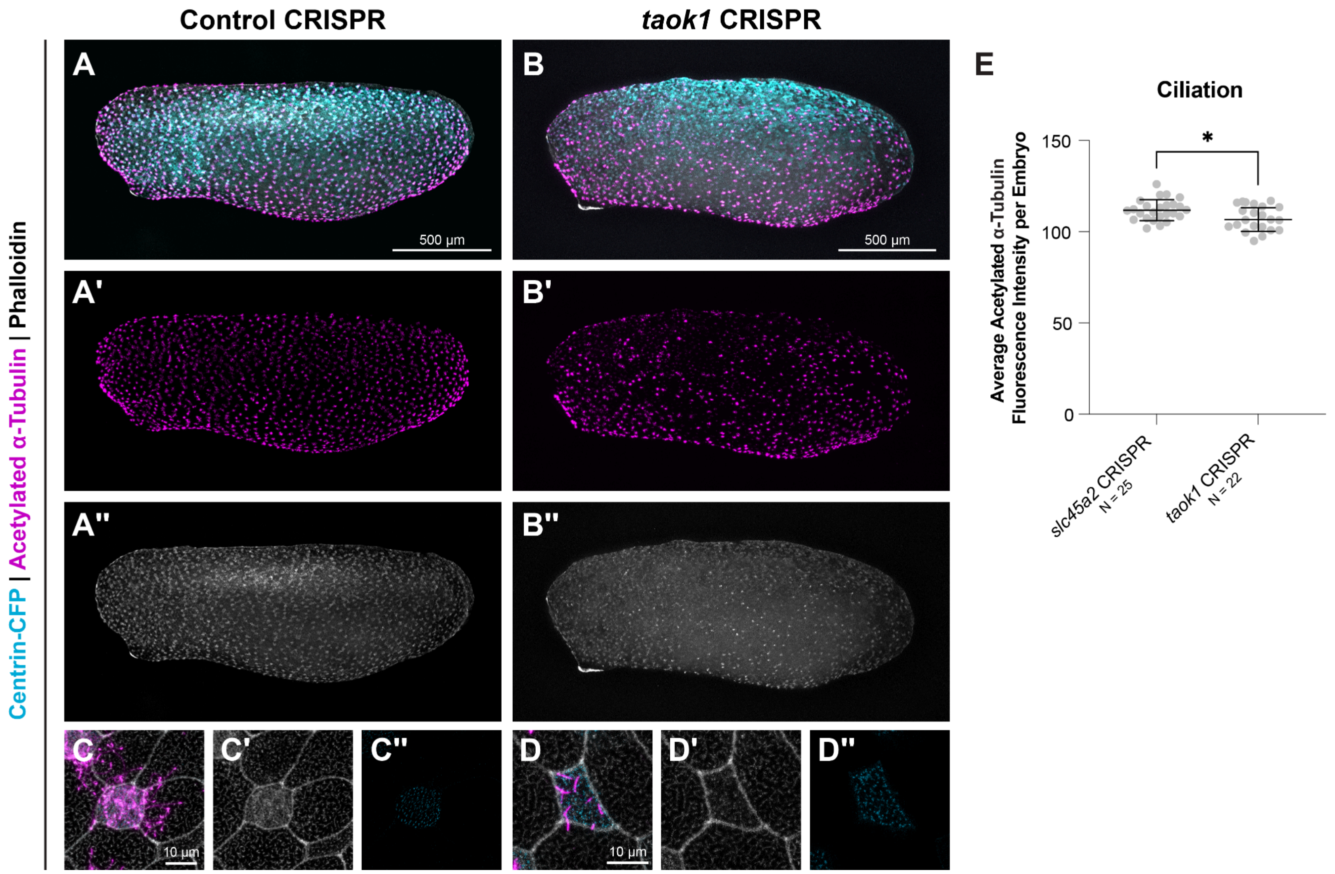

Fig. S7. *taok1* CRISPR loss of function phenocopies *taok1* morpholino loss of function with respect to ciliation. (A - B'') NF stage 28 *X. tropicalis* embryos stained for Acetylated α-Tubulin (magenta), Phalloidin (gray), and injected with Centrin-CFP (blue). *taok1* CRISPR knockdown (B-B'') leads to a reduction in ciliation as seen by a decrease in Acetylated α-Tubulin staining compared to control CRISPR against pigmentation gene *slc45a2*. (C - D''') Images of individual multiciliated cells (MCCs) from Control and *taok1* knockdown conditions. CRISPR-based disruption of *taok1* (D - D'') results in ciliogenesis phenotypes, reduced phalloidin staining (D'), and disrupted basal body distribution (D'') similar to what was observed with the morpholino knockdown of *taok1*. (E) Quantification of Acetylated α-Tubulin staining per embryo. After passing for normality, a two-tailed unpaired t test with welch's correction was performed to compare the *taok1* CRISPR (N = 25) to the Control CRISPR (N = 22), $p < 0.0058$.

Development • Supplementary information

Table S1. List of screened genes (Log2FC, PVal, ASD-satterstrom, CHD-Jin, ASD-CHD Rosenthal, ASD-CHD Genetic, CHD-SFARI).

Available for download at
https://journals.biologists.com/dev/article-lookup/doi/10.1242/dev.204295#supplementary-data

Table S2. List of significant genes input for k-means clustering (per replicate, Cluster number, Log2FC, PVal, Category (ASD, CHD, ASD-CHD)). *Legend: Dx = day number; Rx = replicate number

Available for download at
https://journals.biologists.com/dev/article-lookup/doi/10.1242/dev.204295#supplementary-data

Table S3. All significant (FDR < 0.05) ToppGene Enrichments (Biological Process and Cellular Component) of Cluster 1 genes with CRISPRi screen genes used as background correction.

Available for download at
https://journals.biologists.com/dev/article-lookup/doi/10.1242/dev.204295#supplementary-data

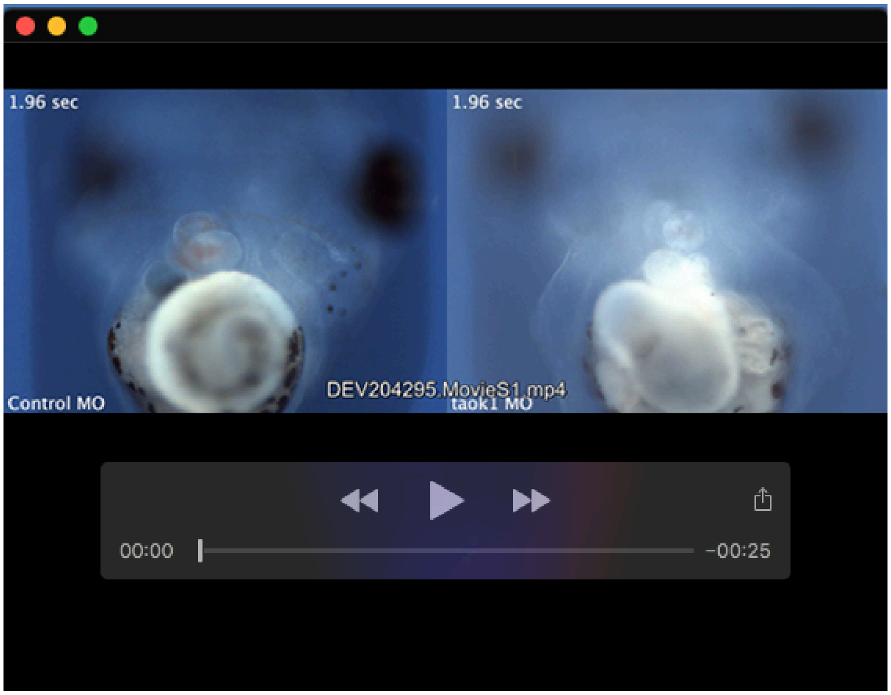

Movie 1. *taok1* knockdown in *X. tropicalis* results in smaller heart ventricle size as shown in live beating hearts.
