# Supplementary material for: Ciliary biology intersects autism and congenital heart disease
Source: Development. 2025 Jun 24;152(12):dev204295. doi: 10.1242/dev.204295 (PMC12273630; doi:10.1242/dev.204295)
Supplement: Supplementary file 1 [file develop-152-204295_review_history.pdf]

Ciliary biology intersects autism and congenital heart disease

Nia Teerikorpi, Kate E. McCluskey, Ethel Bader, Micaela C. Lasser, Sheng Wang, Catherine H. Nguyen, James D. Schmidt, Elina Kostyanovskaya, Nawei Sun, Jeanselle Dea, Tomasz J. Nowakowski, A. Jeremy Willsey and Helen Rankin Willsey
DOI: 10.1242/dev.204295

Editor: Francois Guillemot

Review timeline

Original submission:	31 July 2024
Editorial decision:	28 August 2024
First revision received:	11 February 2025
Editorial decision:	20 March 2025
Second revision received:	17 April 2025
Accepted:	23 April 2025

Original submission

First decision letter

MS ID#: dev.204295

MS TITLE: Ciliary biology intersects autism and congenital heart disease

AUTHORS: Nia Teerikorpi; Micaela C Lasser; Sheng Wang; Elina Kostyanovskaya; Ethel Bader; Nawei Sun; Jeanselle Dea; Tomasz J. Nowakowski; A. Jeremy Willsey; Helen Rankin Willsey

Dear Dr Willsey,

I have now received the reports of three referees on your manuscript, and I have reached a decision. The referees' comments are appended below, or you can access them online: please go to:

As you will see, the referees express great interest in your work, but they also have some significant criticisms and recommend a substantial revision of your manuscript before we can consider publication. In particular they request that you investigate the mechanisms underlying the phenotypes observed, e.g. with an analysis of neural precursor proliferation and survival, and that you control for the specificity of the knockdown phenotypes. If you are able to revise the manuscript along the lines suggested, which may involve further experiments, I will be happy to receive a revised version of the manuscript. Your revised paper will be re-reviewed by the original referees, and acceptance of your manuscript will depend on your addressing satisfactorily their major concerns. Please also note that Development will normally permit only one round of major revision.

If it would be helpful, you are welcome to contact us to discuss your revision in greater detail. Please send us a point-by-point response indicating your plans for addressing the referees' comments, and we will look over this and provide further guidance.

Please attend to all of the reviewers' comments and ensure that you clearly highlight all changes made in the revised manuscript. Please avoid using 'Tracked changes' in Word files as these are lost in PDF conversion. I should be grateful if you would also provide a point-by-point response detailing

© 2025. Published by The Company of Biologists under the terms of the Creative Commons Attribution License (https://creativecommons.org/licenses/by/4.0/).

how you have dealt with the points raised by the reviewers in the 'Response to Reviewers' box. If you do not agree with any of their criticisms or suggestions please explain clearly why this is so.

Reviewer 1

Advance summary and potential significance to field

This manuscript describes the identification of genes likely to confer risk for both ASD and congenital heart disease (CHD) and provides evidence that this subset of genes is enriched for roles in ciliary biology. Specifically, the authors use a CRISPRi screen of 361 ASD, CHD, and ASD-CHD genes in human NPCs and identify a subset of 145 genes that impact NPC proliferation, which may represent a subgroup associated with shared risk, given the association of ASD genes and altered neuronal proliferation. Next, the authors cluster 54 of the genes with the largest fold-change in sgRNA representation and identify four clusters with shared effects on proliferation. They find that the cluster with the most significant enrichment of StringDB interactions is enriched for ciliary biology and demonstrate that the disruption of seven of these genes impacts the percentage of ciliated cells (RPE1, NPCs) in vitro as well as cilia length. Finally, they show that knockdown of one of these genes, which is expressed in ciliary bodies, TAOK1, in Xenopus, affects both brain and heart development, suggesting that the genes identified in this study are relevant for the development of both systems.

Overall, this study is impactful in that it addresses a central question of how to subgroup ASD genes in biologically meaningful ways and provides new evidence that a subset of these genes, particularly those involved in ASD and CHD risk, are involved in ciliary biology.

Comments for the author

1. One major concern involves the use of morpholinos to knockdown TAOK1 in Xenopus (Figure 4) and the lack of validation provided for the method. The gold standard for gene disruption in model systems is CRISPR, where CRISPR/Cas9-induced targeted disruption of the gene can be validated. CRISPR/Cas9 was previously used by this group to target multiple ASD and CHD genes in Xenopus (Willsey et al. 2021, Rosenthal et al. 2021), so it is not clear why morpholinos were used in these experiments. A major drawback to morpholinos is their potential for off-target effects, such that recommendations for confirming morpholino-induced phenotypes include (i) validating phenotypes using a second morpholino (e.g. splice-blocking and translation-blocking); and (ii) demonstrating rescue of the phenotype(s) by reintroducing the wild-type mRNA, yet these were not shown in this study. In addition, no validation of taok1 knockdown is provided (e.g. western blot). It is important to validate the specificity of the phenotypes, particularly because they involve decreased size of the brain and heart, which may be more susceptible to off-target effects. Validation of the phenotypes using CRISPR/Cas9 alone would also be acceptable.

2. The authors suggest that the subset of CHD genes that impact NPC proliferation may represent shared CHD and ASD risk. One question is whether there is any data from the ASD literature to give additional support to this finding (this was mentioned for TAOK1). Also, the study found that ~40% of the genes in all categories, including ASD only, CHD only, and shared ASD-CHD genes, are involved in NPC biology. This suggests that there may be a subset of genes associated with developmental disorders more broadly that impact progenitor proliferation and may not be specific to the overlap of ASD and CHD. It may be useful for the authors to comment on the specificity of this finding (e.g. whether there other risk gene datasets that do not show this enrichment).

Minor-

1. The authors state a few times that the genes they identified are "required" for ciliary biology (e.g. page 5, line 166; page 7, line 203), yet it may be more accurate to say that the experiments show that loss of function of these genes "impacts" cilia development (i.e., "required" suggests that cilia cannot form without these proteins, which may have a range of different roles).

2. The GO enrichment analysis is mentioned in the main text but is not clearly described in the Methods.

© 2025. Published by The Company of Biologists under the terms of the Creative Commons Attribution License (https://creativecommons.org/licenses/by/4.0/).

3. It would be helpful to add the total number of genes included in the analysis to the Venn diagram in Fig. 1A. Also, it might be helpful to show a combined Venn diagram (as opposed to the separate circles at the bottom of the figure, which is unclear).

4. I would suggest adding in the main text the difference between Fig. 3 (normalized data) and Fig. S4 (non-normalized data). This was mentioned in the Methods but not clear in the main text.

5. Page 1, lines 34-35 and Page 3, lines 72-73 are not complete sentences.

Reviewer 2

Advance summary and potential significance to field

The authors seek to address the question of congenital heart disease (CHD) and autism spectrum disorder (ASD). Recent genetic studies have found an interesting overlap between ASD and CHD risk genes suggesting that the co-occurrence of these diseases may be explained by the roles of these genes both in heart and brain development - as opposed to what is commonly assumed that ASD in CHD patients is due to acquired complications of CHD either fetally, prenatally, or peri-operatively.

To address this question, the authors utilized neural progenitor cells to interrogate the potential role of congenital heart disease candidate genes on neurogenesis. Using CRISPRi in NPCs, 361 genes (ASD, CHD, and ASD-CHD shared) were disrupted to identify 145 total genes that significantly impacted survival and/or proliferation of NPCs, which was further restricted to 54 genes with a greater than 1.5x fold change in at least one timepoint. Clustering and GOterm analysis revealed ciliogenesis regulators as highly enriched in the first cluster, which also had the highest number of interactions within the cluster. Focusing on the seven ASD-CHD shared genes within this cluster, knockdown in RPEs revealed defects in the rate of ciliated cells and the length of the cilia that were present. Knockdown in NPCs of three of the seven genes affects ciliogenesis and Arl13b expression. Narrowing further to TAOK1, experiments in Xenopus laevis reveal multiciliated cell localization in the epidermis, and a role in epidermal ciliogenesis upon knockdown in tropicalis. Telencephalon size decreased upon knockdown in tropicalis, as well as decreases in ventricle size.

Though this study is thorough, interesting, and relevant to both brain and heart development, as it stands, this manuscript is not suitable for Development. Per instructions to the reviewer, "paper should make a significant and novel contribution to our understanding of developmental mechanisms." Unfortunately, this paper fails this test and therefore is not suitable for Development. I have not considered this paper in the "Techniques or Resources" section. If this were a consideration, then there is a tremendous amount of data that needs to be included in the paper that is currently lacking (complete GO terms for all clusters, tables of all the genes including excluded genes, etc - none of the Tables were available to this reviewer)

Fundamentally, a mechanism for cardiac development and neurogenesis is lacking. Arguing that cilia affect heart and brain development is not novel - there are litterally hundreds of papers that state this from Shh patterning of the neural tube to the entire field of left-right patterning. While TAOK1 is a novel cilia gene (and therefore is very interesting), no mechanism is presented that would meet the bar for Development.

Ciliogenesis can be affected through multiple mechanisms (changes in proliferation, cilia growth, ciliary maintenance, etc), and without similar analysis (subcellular localization and knockdown experiments at an organism level) performed on the other 6 genes in Cluster 1, it is unclear whether this data defines a clear pathway to better characterization of ASD/CHD overlap. The other 3 clusters also don't show similar network enrichments and were not analyzed for common biological processes. Additionally, the link between mulitciliated cell localization on the epidermis and structural heart malformations is unclear.

Comments for the author

© 2025. Published by The Company of Biologists under the terms of the Creative Commons Attribution License (https://creativecommons.org/licenses/by/4.0/).

Below we provide some comments which we hope the authors may find useful as they prepare this manuscript for another journal:

Do you think the enrichment in sgRNA presence in NPCs in your screen is due to changes in proliferation? Did/can you measure proliferation? I wonder if the reduction in ciliogenesis is secondary to changes in the cell cycle as opposed to cilia based signaling.

Using arl13b for cilia in RPE1 and NPCs (Figure 3): could the reduction in arl13b signal just be an arl13b localization defect? Are those cilia intact but exhibit defects in arl13b localization? An additional marker for cilia could be helpful here. In the cilia field, to state that cilia are lost exclusively using a single marker is not convincing.

TAOK1 MO in Xenopus (Figure 4) - evidence of knockdown or specificity? - (only one MO is tested and no rescue or CRISPR based gene depletion to show specificity). How much is TAOK1 expression reduced in your experimental conditions (low dose MO for epidermis versus high dose MO for brain and heart morphology)? No looping defects for TAOK1 MO (assume as much since nothing was reported) which is a bit odd given the role of cilia in LR patterning? Also, depending on whether the heart is in systole or diastole, ventricle size can vary wildly (as seen by your quantification). Were embryos anesthetized before fixation? Since these are not volumetric measurements, it might be more accurate to take videos of unstained hearts (in anesthetized animals just after anesthesia application) in systole and diastole and measure the area of the heart in both phases to determine whether the actual size is disrupted.

Finally, if the authors wish to consider this paper in the Techniques and Resources section, (I think it could be) but would require significant reformatting to identify genes in different classes that the community could investigate.

Reviewer 3

Advance summary and potential significance to field

In this manuscript, Teerikorpi et al describe a the use of a CRISPRi screen to identify genes that have differentially abundant targeting guides during neuroprogenitor differentiation. The ability to predict risk for autism spectrum disorders is an important goal in developmental medicine. Given the increased prevalence of autism spectrum disorders among people with congenital heart disease, known human congenital heart disease risk genes are a good place to start. However, their model seems to be replicate homozygous loss-of-function genotypes, while their gene list contains genes that function via an autosomal dominant phenotype and/or gain-of-function mechanisms. Further, their in vivo model characterizes primarily organ size and not function or mechanisms of development. These limitations reduce the potential impact of their work in an important field.

Comments for the author

Scientific comments:

- The authors claim to be testing the hypothesis that congenital heart disease (CHD) genes alter neurogenesis, and that leads to autism spectrum disorder (ASD) risk. However, all that was assayed was relative abundance of CRISPRi guides, which was used to infer impact on cellular proliferation/survival. For example, the claim to have demonstrated neurogenesis phenotypes with similar trajectories in line 104 is a strong overinterpretation. Given the lack of log2FC cutoffs to generate the genes discussed in Figure 2, it is unclear how many genes really had a differential accumulation of guides that would be potentially biologically meaningful, and whether any observed differences in cell proliferation/survival were unique to neurons. It is possible that the strong inhibition of many genes could have a universal impact on early progenitor cells and therefore potentially associated with early lethality (most of the CHD genes are heterozygous genotypes with homozygosity presumed to be associated with early demise; the pLI/LOEUF values from gnomAD for much of your list will demonstrate genetic constraint). To improve the first part of the hypothesis (that neurogenesis was altered) it would be useful to many ASD genes assayed had an NPC survival phenotype in their assay. What is the sensitivity and specificity of this assay for the

© 2025. Published by The Company of Biologists under the terms of the Creative Commons Attribution License (https://creativecommons.org/licenses/by/4.0/).

known genes that were studied? It must also be considered that differences in neuronal differentiation that do not impact proliferation/survival would not be ascertained by this assay. Further, it remains unclear how the neuronal progenitor cell (NPC) phenotypes observed in the in vitro assay may related to risk for ASD, so the authors should be clear that they did not test the second portion of their hypothesis in this work.

- The screen seems designed to replicate homozygous loss-of-function genotypes. This could potentially explain the enrichment for cilia genes, which are a significant portion of known human autosomal recessive CHD risk genes. It may be useful to limit the tested genes, both ASD and CHD, to just those that operate by this mechanism. Otherwise the gene mechanisms do not match human disease risk and therefore relevance to human genetic disease could be overinterpreted. This relates to the important question of how the ASD/CHD gene lists were obtained. Were these the curated gene lists published by the references manuscripts, or a further curation of those lists?
- Overall the inclusion of genes regardless of impact (log2FC) in Figure 2 limits its interpretation, as does the use of an overly generous FDR of 0.1. Results with FDR <0.05 should be provided. It would be helpful to have information about the number of guides detected per gene, and the number of reads per guide, to be able to assess the screen performance. Quantification of guide abundance between replicates should be provided to demonstrate the confidence in differential abundance calculations. Similarly, p-values should be added to Figure 2D. Comparison with all genes is irrelevant given the functional enrichment in the curated gene set and would only be appropriate if a genome-wide candidate gene screen had been performed.
- Morpholino technology is prone to off-target effects. It also seems that the injection itself may have been damaging: what was the proportion of moderate disruption to cilial development in uninjected embryos? 19% reported for the control MO is a high background. Further, cilia was imaged in static form and function was not assessed. Finally, data were expressed at percentage of MCCs - what about absolute numbers of MCCs by phenotype per embryo? Would be helpful to see variants between treatments as well as among embryos. Finally, the phenotypes of altered size at one developmental point is not a strong proxy for ASD and CHD.

Other comments:

- The 6th affiliation is missing a comma
- In line 49, would be more balanced to say "ASD and CHD likely share some common biology" as there are many genetic causes for either disorder where only one or the other diagnosis is present
- It is not clear that a new sentence was intended to be started in line 72 - it makes more sense as a subordinate clause of the earlier sentence
- Selection of non-targeting controls was not described
- In line 166 is not fully accurate to call them ASD-CHD shared risk genes. As pointed out in the next section, KMT2E and TAOK1 have not been associated with human CHD. Could call them candidate ASD-CHD shared risk genes
- It would be helpful to clarify in Methods if BFP screening was used to select cells for harvesting at all timepoints. Were dead cells removed?
- K values in Figure S1 should also be compared using cross-validation error to determine the optimal number of groups in the k-means clustering analysis
- Normalization in Figure 3 should be explained clearly to be able to interpret the differences with Figure S4
- Costained images of hTAOKA-GFP cells, wildtype, and hTAOK1 transfected cells without GFP should be included in Figure S5. Otherwise it is unclear if overexpression of TAOK1 may lead to more fundamental changes in cilia formation
- Reporting participants from DECIPHER should be unbiased - how many total participants with de novo variants in TAOK1 were identified, and what proportion of them had CHD? Did any of them have other potential causal variants? Given that the Xenopus model had near complete deletion of Taok1, are you proposing a dominant negative mechanism for human disease?

First revision

<u>Author response to reviewers' comments</u>

© 2025. Published by The Company of Biologists under the terms of the Creative Commons Attribution License (https://creativecommons.org/licenses/by/4.0/).

Response to Reviewers

We genuinely appreciate the feedback received and the care with which you all have reviewed this manuscript. Your comments have greatly improved it. We have addressed your comments below in blue.

REVIEWER 1: SUMMARY OF THE ADVANCE MADE IN THIS PAPER AND ITS POTENTIAL SIGNIFICANCE TO THE FIELD

This manuscript describes the identification of genes likely to confer risk for both ASD and congenital heart disease (CHD) and provides evidence that this subset of genes is enriched for roles in ciliary biology. Specifically, the authors use a CRISPRi screen of 361 ASD, CHD, and ASD-CHD genes in human NPCs and identify a subset of 145 genes that impact NPC proliferation, which may represent a subgroup associated with shared risk, given the association of ASD genes and altered neuronal proliferation. Next, the authors cluster 54 of the genes with the largest fold-change in sgRNA representation and identify four clusters with shared effects on proliferation. They find that the cluster with the most significant enrichment of StringDB interactions is enriched for ciliary biology and demonstrate that the disruption of seven of these genes impacts the percentage of ciliated cells (RPE1, NPCs) in vitro as well as cilia length. Finally, they show that knockdown of one of these genes, which is expressed in ciliary bodies, TAOK1, in Xenopus, affects both brain and heart development, suggesting that the genes identified in this study are relevant for the development of both systems. Overall, this study is impactful in that it addresses a central question of how to subgroup ASD genes in biologically meaningful ways and provides new evidence that a subset of these genes, particularly those involved in ASD and CHD risk, are involved in ciliary biology.

We appreciate the time you have taken to thoroughly read our manuscript and your thoughtful summary of our findings.

SUGGESTIONS TO AUTHORS

1. One major concern involves the use of morpholinos to knockdown TAOK1 in Xenopus (Figure 4) and the lack of validation provided for the method. The gold standard for gene disruption in model systems is CRISPR, where CRISPR/Cas9-induced targeted disruption of the gene can be validated. CRISPR/Cas9 was previously used by this group to target multiple ASD and CHD genes in Xenopus (Willsey et al. 2021, Rosenthal et al. 2021), so it is not clear why morpholinos were used in these experiments. A major drawback to morpholinos is their potential for off-target effects, such that recommendations for confirming morpholino-induced phenotypes include (i) validating phenotypes using a second morpholino (e.g. splice-blocking and translation-blocking); and (ii) demonstrating rescue of the phenotype(s) by reintroducing the wild-type mRNA, yet these were not shown in this study. In addition, no validation of taok1 knockdown is provided (e.g. western blot). It is important to validate the specificity of the phenotypes, particularly because they involve decreased size of the brain and heart, which may be more susceptible to off-target effects. Validation of the phenotypes using CRISPR/Cas9 alone would also be acceptable.

We appreciate this comment and have now validated the role of TAOK1 in cilia formation in *Xenopus* in two orthogonal ways: first, we now include a CRISPR phenocopy (**Fig. S7**) that shows the same cilia phenotype; second, we now show that a commercial TAOK1 antibody shows the same localization pattern on cilia as the GFP overexpression (**Fig. S6A-C**), and this staining is greatly reduced by the TAOK1 morpholino (**Fig. S6D-F**). Together, we think these two lines of evidence bolster our initial findings and give sufficient evidence that the results are indeed due to TAOK1 loss of function.

2. The authors suggest that the subset of CHD genes that impact NPC proliferation may represent shared CHD and ASD risk. One question is whether there is any data from the ASD literature to give additional support to this finding (this was mentioned for TAOK1). Also, the study found that ~40% of the genes in all categories, including ASD only, CHD only, and shared ASD-CHD genes, are involved in NPC biology. This suggests that there may be a subset of genes associated with developmental disorders more broadly that impact progenitor proliferation and may not be specific

© 2025. Published by The Company of Biologists under the terms of the Creative Commons Attribution License (https://creativecommons.org/licenses/by/4.0/).

to the overlap of ASD and CHD. It may be useful for the authors to comment on the specificity of this finding (e.g. whether there are other risk gene datasets that do not show this enrichment).

In our manuscript we have acknowledged a well-replicated link between NPC proliferation and ASD for many genes **(LINES 61-64): "***In vivo* and *in vitro* studies have repeatedly identified neural progenitor cell (NPC) proliferation as a convergent phenotype in ASD (Sun et al. 2024; H. R. Willsey et al. 2022, 2021; Sacco, Cacci, and Novarino 2018; Packer 2016; Iakoucheva, Muotri, and Sebat 2019; Courchesne et al. 2019; Marchetto et al. 2017; Lalli et al. 2020)". As for the specific question of whether CHD genes with shared risk for ASD are more likely to impact NPC proliferation–that is precisely the question that we aimed to address here. With respect to the observation that disruption of ~40% of genes in all groupings disrupts NPC biology, we would like to note that ASD "only" and CHD "only" genes may actually carry shared risk not yet identified by contemporary genetic studies, as gene discovery is not yet saturated (i.e. some or many of these may actually be ASD-CHD genes). Thus, we still hypothesize that stratifying CHD genes based on their impact on proliferation of NPCs will be valuable in prioritizing CHD genes with the greatest likelihood of shared risk with ASD, regardless of whether they are initially classified as CHD only or ASD-CHD. Either way, we agree with the reviewer that our work does not provide evidence that disruption of NPC proliferation is specific to ASD and/or CHD genes. Indeed, we believe this raises interesting questions about other conditions that have neurodevelopmental comorbidities. We have added a statement to the discussion in order to clarify this point (**LINES: 253-254**): "Our work does not provide evidence that disruption of NPC proliferation is specific to ASD and/or CHD genes."

Minor-

1. The authors state a few times that the genes they identified are "required" for ciliary biology (e.g. page 5, line 166; page 7, line 203), yet it may be more accurate to say that the experiments show that loss of function of these genes "impacts" cilia development (i.e., "required" suggests that cilia cannot form without these proteins, which may have a range of different roles).

In the revised manuscript we have now changed "required" to "impacts" wherever we used this phrasing.

2. The GO enrichment analysis is mentioned in the main text but is not clearly described in the Methods.

Thank you for catching this oversight. In the revised manuscript we have now included a clear description of the GO enrichment analysis (**LINES 350-352**): "We performed gene ontology enrichment analysis of the Cluster 1 genes with ToppGene (J. Chen et al. 2009), using the 361 genes from our CRISPRi library as background. We considered gene ontology terms with Benjamini-Hochberg FDR < 0.05 as significantly enriched and calculated fold-enrichment using the following formula: (Hit Count in Input Genest/Hit Count in Background)/(Number of Input Genes/Number of Background Genes)."

3. It would be helpful to add the total number of genes included in the analysis to the Venn diagram in Fig. 1A. Also, it might be helpful to show a combined Venn diagram (as opposed to the separate circles at the bottom of the figure, which is unclear).

We have edited this schematic to be more clear regarding the total number of genes in each category. We have moved the specific details regarding the sources for evidence of risk to the figure legend and methods.

4. I would suggest adding in the main text the difference between Fig. 3 (normalized data) and Fig. S4 (non-normalized data). This was mentioned in the Methods but not clear in the main text.

We have now added a description of the difference between Figure 3 (Normalized) and Figure S4 (non-normalized) into the main text **(LINES 140-141)**: "We measured percent ciliated cells and cilia length (**Fig. S3B-C**) and then normalized these data based on average BFP+ cell density of the non-targeting control sgRNA 1 (**Fig. 3**)."

5. Page 1, lines 34-35 and Page 3, lines 72-73 are not complete sentences.

© 2025. Published by The Company of Biologists under the terms of the Creative Commons Attribution License (https://creativecommons.org/licenses/by/4.0/).

Thank you for catching this error. We have edited the phrasing on Page 1, lines 30-31 (**NOW LINES 31-33**) to “In vivo investigation of TAOK1 in Xenopus tropicalis reveals a role for the gene in motile cilia formation and heart development, supporting its prediction as a CHD gene.” For lines 72-73 (**NOW LINES 70-73**), it now reads: “These results not only identify a set of CHD genes that are likely associated with an increased likelihood of ASD but also suggest that cilia play a significant role in the shared biological mechanisms of both disorders, consistent with recent findings and ciliary biology to ASD (Willsey *et al.*, 2022; Sun *et al.*, 2024; Kostyanovskaya *et al.*, 2025).

REVIEWER 2: SUMMARY OF THE ADVANCE MADE IN THIS PAPER AND ITS POTENTIAL SIGNIFICANCE TO THE FIELD

The authors seek to address the question of congenital heart disease (CHD) and autism spectrum disorder (ASD). Recent genetic studies have found an interesting overlap between ASD and CHD risk genes suggesting that the co-occurrence of these diseases may be explained by the roles of these genes both in heart and brain development - as opposed to what is commonly assumed that ASD in CHD patients is due to acquired complications of CHD either fetally, prenatally, or peri-operatively. To address this question, the authors utilized neural progenitor cells to interrogate the potential role of congenital heart disease candidate genes on neurogenesis. Using CRISPRi in NPCs, 361 genes (ASD, CHD, and ASD-CHD shared) were disrupted to identify 145 total genes that significantly impacted survival and/or proliferation of NPCs, which was further restricted to 54 genes with a greater than 1.5x fold change in at least one timepoint. Clustering and GOterm analysis revealed ciliogenesis regulators as highly enriched in the first cluster, which also had the highest number of interactions within the cluster. Focusing on the seven ASD-CHD shared genes within this cluster, knockdown in RPEs revealed defects in the rate of ciliated cells and the length of the cilia that were present. Knockdown in NPCs of three of the seven genes affects ciliogenesis and Arl13b expression. Narrowing further to TAOK1, experiments in Xenopus laevis reveal multiciliated cell localization in the epidermis, and a role in epidermal ciliogenesis upon knockdown in tropicalis. Telencephalon size decreased upon knockdown in tropicalis, as well as decreases in ventricle size.

We appreciate this thorough summary of the findings laid out in the manuscript.

Though this study is thorough, interesting, and relevant to both brain and heart development, as it stands, this manuscript is not suitable for Development. Per instructions to the reviewer, "paper should make a significant and novel contribution to our understanding of developmental mechanisms." Unfortunately, this paper fails this test and therefore is not suitable for Development. I have not considered this paper in the "Techniques or Resources" section. If this were a consideration, then there is a tremendous amount of data that needs to be included in the paper that is currently lacking (complete GO terms for all clusters, tables of all the genes including excluded genes, etc - none of the Tables were available to this reviewer). Fundamentally, a mechanism for cardiac development and neurogenesis is lacking. Arguing that cilia affect heart and brain development is not novel - there are literally hundreds of papers that state this from Shh patterning of the neural tube to the entire field of left-right patterning. While TAOK1 is a novel cilia gene (and therefore is very interesting), no mechanism is presented that would meet the bar for Development. Ciliogenesis can be affected through multiple mechanisms (changes in proliferation, cilia growth, ciliary maintenance, etc), and without similar analysis (subcellular localization and knockdown experiments at an organism level) performed on the other 6 genes in Cluster 1, it is unclear whether this data defines a clear pathway to better characterization of ASD/CHD overlap. The other 3 clusters also don't show similar network enrichments and were not analyzed for common biological processes. Additionally, the link between multiciliated cell localization on the epidermis and structural heart malformations is unclear.

We are grateful to the reviewer for encouraging us to better clarify the novelty of this work and put it into context with the existing literature. We regret that the way we wrote the manuscript made it seem like we were claiming novelty for the relationship between ciliary biology and heart/brain development. Indeed, this is well-established.

Rather, the novelty of our work lies in the finding that the comorbidity between CHD and ASD is related to ciliary biology, with direct evidence from well-supported risk genes identified from patient studies. Prior to this work, while the genetic overlap between the disorders was

© 2025. Published by The Company of Biologists under the terms of the Creative Commons Attribution License (https://creativecommons.org/licenses/by/4.0/).

established, the molecular basis of the ASD/CHD comorbidity was unknown and relatively unexplored. Here we have established that ciliary biology is at the intersection of these shared risk genes and may underlie this comorbidity. Further, while the essential role of cilia in brain development is clear, the finding that ciliary biology is relevant to many ASD risk genes is novel, and can provide new therapeutic avenues. We have now added the following statement to the discussion to better articulate these distinctions (**LINES 217-219**): “This work provides the first direct evidence that ciliary biology underlies the comorbidity between ASD and CHD, establishing a molecular basis for this overlap and highlighting cilia as a novel point of convergence for shared risk genes.”

Regarding mechanism, deep mechanistic understanding of our seven genes of interest was unfortunately outside of the scope of this paper. Rather, we chose instead to validate the relevance of one of them in ciliary biology *in vivo*. In the revised manuscript, we have now added additional experiments on TAOK1 in *Xenopus*. We now show its subcellular localization at basal bodies and ciliary axonemes by both GFP overexpression (**Fig. S6A-B**) and by endogenous antibody staining (**Fig. S6C**). We also validate this antibody in *X. tropicalis* (**Fig. S6D-F**). We now also provide a CRISPR phenocopy in *tropicalis* to complement the morpholino work (**Fig. S7 A-C**). Regarding the specific mechanism by which TAOK1 affects ciliogenesis, we now show there is a basal body docking defect and an apical actin organization defect (**Fig. 4C-D,F**). Further, it is known that TAOK1 affects microtubule stability (Byeon and Yadav 2024), and we have added this point in the discussion regarding its molecular mechanism in ciliary biology, since defects in microtubule stability impair cilia formation and basal body docking (**LINES 222-225**). Of course, extensive future work is needed for each of these genes to fully understand the mechanisms by which they influence cilia biology, but we feel the conceptual advances we have made are novel and open up many new research opportunities for each gene.

Regarding the link between epidermal multiciliated cells and heart development: we were using the epidermis as a model of motile cilia biology given its technical advantages experimentally over the gastrocoel roof plate and its established use as a powerful model for studying CHD genes. We have clarified this point now in the results (**LINES 171-174**): “To do this, we used the multiciliated Xenopus epidermis as a model for motile cilia biology due to its accessibility, experimental tractability, and established relevance for studying CHD genes (Garfinkel and Khokha, 2017; Kulkarni et al., 2018).”

SUGGESTIONS TO AUTHORS

Below we provide some comments which we hope the authors may find useful as they prepare this manuscript for another journal:

Do you think the enrichment in sgRNA presence in NPCs in your screen is due to changes in proliferation? Did/can you measure proliferation? I wonder if the reduction in ciliogenesis is secondary to changes in the cell cycle as opposed to cilia based signaling.

Changes in sgRNA abundance could be caused by multiple issues, including proliferation, survival, and differentiation, so it is certainly possible that proliferation is changed and that reduction in ciliation is secondary to cell cycle changes and we now mention this in the discussion (**LINES 245-250**): “As our screen cannot disentangle impacts on proliferation, differentiation, or survival, it is difficult to determine which process(es) is the cause of the shared phenotype. Further, the screen likely misses genes that would show phenotypes during differentiation, but not during proliferation. While many ASD gene variants cause NPC proliferation defects, it also remains unclear how the observed NPC phenotypes relate to ASD risk directly.”

However, in our ciliary phenotyping in RPE-1s, the cells were serum-starved, so they were not actively cycling, and ciliary phenotypes were still observed. We cannot serum-starve NPCs (they die), so we grew them at high density to mitigate this issue. Nevertheless, to address the proliferation issue, we now add new data showing a cell cycle analysis for *TAOK1* knockdown in NPCs (**Fig. S5**). We observe that knock-down of *TAOK1* does not change cell cycle progression (**Fig. S5**), so at least for this gene the ciliation defects do not seem to be secondary to cell cycle issues in this context.

© 2025. Published by The Company of Biologists under the terms of the Creative Commons Attribution License (https://creativecommons.org/licenses/by/4.0/).

Using arl13b for cilia in RPE1 and NPCs (Figure 3): could the reduction in arl13b signal just be an arl13b localization defect? Are those cilia intact but exhibit defects in arl13b localization? An additional marker for cilia could be helpful here. In the cilia field, to state that cilia are lost exclusively using a single marker is not convincing.

It is possible that some of the phenotypes we observe are due to localization defects of ARL13B, particularly since ARL13B intensity within cilia changes for several conditions. We now explicitly acknowledge this possibility in the discussion (**LINES 225-227**): "Additionally, we observed changes to ARL13B signal intensity in several of the *in vitro* conditions. Future work should explore whether each perturbation causes changes in ciliary trafficking and/or structure and how these changes affect ciliary signaling and fluid flow." For TAOK1, we also show acetylated-alpha tubulin as a ciliary marker in *Xenopus*, showing a reduction in cilia number as well as an accompanying basal body docking and actin organization defect (**Fig. 4A-F**), so at least for this gene it seems to be required for cilia formation and not just ciliary trafficking.

TAOK1 MO in Xenopus (Figure 4) - evidence of knockdown or specificity? - (only one MO is tested and no rescue or CRISPR based gene depletion to show specificity). How much is TAOK1 expression reduced in your experimental conditions (low dose MO for epidermis versus high dose MO for brain and heart morphology)? No looping defects for TAOK1 MO (assume as much since nothing was reported) which is a bit odd given the role of cilia in LR patterning? Also, depending on whether the heart is in systole or diastole, ventricle size can vary wildly (as seen by your quantification). Were embryos anesthetized before fixation? Since these are not volumetric measurements, it might be more accurate to take videos of unstained hearts (in anesthetized animals just after anesthesia application) in systole and diastole and measure the area of the heart in both phases to determine whether the actual size is disrupted.

We appreciate this comment and have now validated the role of TAOK1 in cilia formation in *Xenopus* in two orthogonal ways: first, we now include a CRISPR phenocopy (**Fig. S7**) that shows the same cilia phenotype; second, we now show that a commercial TAOK1 antibody shows the same localization pattern on cilia as the GFP overexpression (**Fig. S6A-C**), and this staining is greatly reduced by the TAOK1 morpholino (**Fig. S6D-F**), suggesting this is a strong knockdown at this dose. Together, we think these additional lines of evidence bolster our initial findings and give sufficient evidence that the results are indeed due to TAOK1 loss of function.

Regarding heart phenotyping, indeed we did not observe significant looping defects. We repeated the heart experiments to image systole and diastole. Indeed, the ventricle size was much reduced in both phases and now we include a video of the heart defects to better represent both systole and diastole (**Movie S1**). In this video you can see that the heart size is strikingly smaller in both phases.

Finally, if the authors wish to consider this paper in the Techniques and Resources section, (I think it could be) but would require significant reformatting to identify genes in different classes that the community could investigate.

We appreciate this suggestion and will discuss it with the editor.

REVIEWER 3: SUMMARY OF THE ADVANCE MADE IN THIS PAPER AND ITS POTENTIAL SIGNIFICANCE TO THE FIELD

In this manuscript, Teerikorpi et al describe the use of a CRISPRi screen to identify genes that have differentially abundant targeting guides during neuroprogenitor differentiation. The ability to predict risk for autism spectrum disorders is an important goal in developmental medicine. Given the increased prevalence of autism spectrum disorders among people with congenital heart disease, known human congenital heart disease risk genes are a good place to start. However, their model seems to replicate homozygous loss-of-function genotypes, while their gene list contains genes that function via an autosomal dominant phenotype and/or gain-of-function mechanisms. Further, their in vivo model characterizes primarily organ size and not function or mechanisms of development. These limitations reduce the potential impact of their work in an important field.

We appreciate the reviewer's feedback and address your issues below.

© 2025. Published by The Company of Biologists under the terms of the Creative Commons Attribution License (https://creativecommons.org/licenses/by/4.0/).

SUGGESTIONS TO AUTHORS

Scientific comments:

- The authors claim to be testing the hypothesis that congenital heart disease (CHD) genes alter neurogenesis, and that leads to autism spectrum disorder (ASD) risk. However, all that was assayed was relative abundance of CRISPRi guides, which was used to infer impact on cellular proliferation/survival. For example, the claim to have demonstrated neurogenesis phenotypes with similar trajectories in line 104 is a strong overinterpretation. Given the lack of log2FC cutoffs to generate the genes discussed in Figure 2, it is unclear how many genes really had a differential accumulation of guides that would be potentially biologically meaningful, and whether any observed differences in cell proliferation/survival were unique to neurons. It is possible that the strong inhibition of many genes could have a universal impact on early progenitor cells and therefore potentially associated with early lethality (most of the CHD genes are heterozygous genotypes with homozygosity presumed to be associated with early demise; the pLI/LOEUF values from gnomAD for much of your list will demonstrate genetic constraint). To improve the first part of the hypothesis (that neurogenesis was altered) it would be useful to many ASD genes assayed had an NPC survival phenotype in their assay. What is the sensitivity and specificity of this assay for the known genes that were studied? It must also be considered that differences in neuronal differentiation that do not impact proliferation/survival would not be ascertained by this assay. Further, it remains unclear how the neuronal progenitor cell (NPC) phenotypes observed in the in vitro assay may relate to risk for ASD, so the authors should be clear that they did not test the second portion of their hypothesis in this work.

We regret that it was unclear that the genes presented in Figure 2 were restricted by absolute log2FC cutoffs (log2FC ≥ 0.582) and p - values ($p < 0.05$). This is explicitly mentioned in the legend and main text now. We have also removed the statement regarding convergent neurogenesis trajectories (previous line 104, current line 105).

We agree that we cannot rule out the potential influences of survival and differentiation on these results, and now explicitly state this in the discussion (**LINES 245-247**): "As our screen cannot disentangle impacts on proliferation, differentiation, or survival, it is difficult to determine which process(es) is the cause of the shared phenotype." Regarding the relationship between NPC proliferation and ASD risk, there is extensive literature linking these two and we have cited it in this manuscript. Nevertheless we have also added an acknowledgement of how it remains unclear how the NPC phenotypes relate to ASD risk and the limitations of this screen for identifying differentiation phenotypes (**LINES 247-250**): "Further, the screen likely misses genes that would show phenotypes during differentiation, but not during proliferation. While many ASD gene variants cause NPC proliferation defects, it also remains unclear how the observed NPC phenotypes relate to ASD risk directly."

- The screen seems designed to replicate homozygous loss-of-function genotypes. This could potentially explain the enrichment for cilia genes, which are a significant portion of known human autosomal recessive CHD risk genes. It may be useful to limit the tested genes, both ASD and CHD, to just those that operate by this mechanism. Otherwise the gene mechanisms do not match human disease risk and therefore relevance to human genetic disease could be overinterpreted. This relates to the important question of how the ASD/CHD gene lists were obtained. Were these the curated gene lists published by the referenced manuscripts, or a further curation of those lists?

Our risk genes, both for CHD and ASD, are derived from lists generated from exome-wide studies of rare, often de novo, likely gene disrupting variants (heterozygous loss of function) (Satterstrom et al. for ASD and Jin et al. to CHD). We chose genes from so-called "hypothesis-naive" whole exome sequencing studies in order to obtain a representative sample of ASD and CHD genes that is not biased by pre-existing hypotheses about the underlying biology. Regardless, we are likely modeling a stronger loss of function than is seen in affected individuals, but this is the case for both gene sets, suggesting that the cilia-related enrichments observed here are not driven by the *a priori* selection of autosomal recessive genes with known cilia involvement. In other words, because these genes were ascertained based on essentially the same criteria, we believe the fact that the ASD and CHD genes identified by exome-wide sequencing studies focused on rare, heterozygous variants

© 2025. Published by The Company of Biologists under the terms of the Creative Commons Attribution License (https://creativecommons.org/licenses/by/4.0/).

intersect cilia biology, like genes identified based on recessive patterns of inheritance, suggests biologically meaningful functional convergence.

The importance of cilia biology in neurodevelopmental and congenital disorders is increasingly recognized. For example, individuals with genetic ciliopathies such as Bardet-Biedl Syndrome and Joubert Syndrome (caused by variants in cilia-specific genes) have been reported to have a high prevalence of autistic features (Kerr et al. 2016; Ozonoff et al. 1999), supporting the possibility that cilia underlie a subset of autism-relevant biology. Further, other known cilia-related disorders are often comorbid with autism, including hydrocephalus, blindness, asthma, and epilepsy.

Similarly, many CHD genes included in our study are already known to be cilia-related and exhibit clear phenotypes even when heterozygous. While our models may represent stronger loss-of-function effects, this does not alter the intrinsic biology of these genes.

Regardless, we agree that we are producing a strong loss of function, and have acknowledged this limitation in the manuscript explicitly now (**LINES 254-256**): "Additionally, our screen created strong loss-of-function perturbations, highlighting the need for future work to use haploinsufficiency models to further elaborate these dose-dependent mechanisms and disentangle these processes." As in many animal studies, it is far easier to identify robust phenotypes in strong loss of function than in haploinsufficiency, so for this work we have opted to take this approach to generate hypotheses to follow up in models where gene dosage can be more precisely considered (ie, germline mutant animals, patient cell lines, etc).

- Overall the inclusion of genes regardless of impact (log2FC) in Figure 2 limits its interpretation, as does the use of an overly generous FDR of 0.1. Results with FDR <0.05 should be provided. It would be helpful to have information about the number of guides detected per gene, and the number of reads per guide, to be able to assess the screen performance. Quantification of guide abundance between replicates should be provided to demonstrate the confidence in differential abundance calculations. Similarly, p-values should be added to Figure 2D. Comparison with all genes is irrelevant given the functional enrichment in the curated gene set and would only be appropriate if a genome-wide candidate gene screen had been performed.

We regret that it was unclear that the genes presented in Figure 2A were restricted by log2FC cutoffs (log2FC >/< 0.582) and p - values (p < 0.05). We have now emphasized this in the text and legend and hope that this greatly reduces your concern. We have added a p - value demarcation on Figure 2D and limited this analysis to the results compared to the background of just screened genes (ie, we removed the analysis using a standard background).

- Morpholino technology is prone to off-target effects. It also seems that the injection itself may have been damaging: what was the proportion of moderate disruption to cilial development in uninjected embryos? 19% reported for the control MO is a high background. Further, cilia was imaged in static form and function was not assessed. Finally, data were expressed at the percentage of MCCs - what about absolute numbers of MCCs by phenotype per embryo? It would be helpful to see variants between treatments as well as among embryos. Finally, the phenotypes of altered size at one developmental point is not a strong proxy for ASD and CHD.

We appreciate this comment and have now validated the role of TAOK1 in cilia formation in *Xenopus* in two orthogonal ways: first, we now include a CRISPR phenocopy (**Fig. S7**) that shows the same cilia phenotype; second, we now show that a commercial TAOK1 antibody shows the same localization pattern on cilia as the GFP overexpression (**Fig. S6A-C**), and this staining is greatly reduced by the TAOK1 morpholino (**Fig. S6D-F**), suggesting this is a strong knockdown even at the lower dose. Together, we think these additional lines of evidence bolster our initial findings and give sufficient evidence that the results are indeed due to TAOK1 loss of function.

Regarding control morpholino issues: the original cilia phenotyping graph had a control morpholino condition where twice as much control morpholino was injected compared to the TAOK1 morpholino, likely causing that issue in the controls. We have repeated all these experiments now with the same dose across conditions, adding in the CRISPR conditions as well, and the controls no longer show these phenotypes.

© 2025. Published by The Company of Biologists under the terms of the Creative Commons Attribution License (https://creativecommons.org/licenses/by/4.0/).

To address your point about cell-to-cell variability and quantitation, during revision we developed an ImageJ macro to automatically quantify fluorescence intensity of the cilia marker (acetylated alpha-tubulin) within injected cells per embryo. So, now this quantification is quantitative (compared to qualitative before) and by embryo rather than by cell (**Fig. 4E and S7E**). This likely underestimates the effect of the knockdown compared to qualitative phenotyping, but we felt it was worth it to have a quantitative metric where the n compared are embryos, rather than cells (so as not to artificially power the analyses). We also show images of entire embryos to better convey the severity of the phenotypes (and the expected mosaicism). We think this has greatly improved the manuscript and appreciate your suggestions very much. With respect to cilia function, we have commented in the discussion that this is needed, as we assume that such a dramatic loss of cilia will disrupt fluid flow, but this needs to be addressed in future work.

Regarding size as a proxy for ASD or CHD: while many genes for both disorders have been shown to cause such phenotypes in model systems, we agree that they are not proxies for these human conditions. Rather, these experiments were aimed to determine whether TAOK1 affects embryonic development of both organs *in vivo*, which is the case. We have edited the manuscript to ensure that we simply state that TAOK1 impacts heart and brain development.

Other comments:

- The 6th affiliation is missing a comma

We have now included a comma.

- In line 49, would be more balanced to say "ASD and CHD likely share some common biology" as there are many genetic causes for either disorder where only one or the other diagnosis is present

This line now reads: "ASD and CHD likely share some common biology."

- It is not clear that a new sentence was intended to be started in line 72 - it makes more sense as a subordinate clause of the earlier sentence

The sentence now reads: “These results not only identify a set of CHD genes that are likely associated with an increased risk for ASD but also suggest that cilia play a significant role in the shared biological mechanisms of both disorders, consistent with recent findings linking tubulin biology to ASD (H. R. Willsey et al. 2022; Sun et al. 2024).”

- Selection of non-targeting controls was not described

We have added this information into the methods (**LINES 302-304**): “To select non-targeting control sgRNAs for this experiment, we used a set of 225 sequences designed by the Kampmann Lab to have significant mismatches to any known genomic target (Tian et al. 2021).”

- In line 166 is not fully accurate to call them ASD-CHD shared risk genes. As pointed out in the next section, KMT2E and TAOK1 have not been associated with human CHD. Could call them candidate ASD-CHD shared risk genes

We have now added ‘predicted’ ahead of “ASD-CHD shared risk genes” in this line.

- It would be helpful to clarify in Methods if BFP screening was used to select cells for harvesting at all timepoints. Were dead cells removed?

We have clarified this methodology now (**Lines 324-331**): “On day 3 we passaged cells using accutase after washing 3 times to remove dead cells. Approximately 1 million cells were fluorescence activated cell sorted (FACS) to ensure ≥85% of cells from each of the 3 replicate conditions were expressing BFP. Then 5 million cells from each replicate (≥85% BFP+) were harvested (Day 0; library representation ~1,000 cells per sgRNA). The remainder of the cells were re-plated onto three 10 cm cell culture dishes for later time points. We seeded 2 million NPCs per plate and cultured in N2/B27 medium as described previously. The cells were passaged every 3-5 days and approximately 5 million cells from each replicate were then harvested at days 5, 10 and 20, after validating ≥85% BFP+ cells via FACS.”

- K values in Figure S1 should also be compared using cross-validation error to determine the

© 2025. Published by The Company of Biologists under the terms of the Creative Commons Attribution License (https://creativecommons.org/licenses/by/4.0/).

optimal number of groups in the k-means clustering analysis

In choosing the number of clusters, we optimized for the highest resolution obtainable while operating within standard heuristics for choosing K. Specifically, we choose four clusters because this is the largest number of clusters supported by both an elbow plot and the Silhouette method (now mentioned in the main text and now shown in **Fig. S1**). Additionally, as methods for determining the optimal number of clusters have a degree of subjectivity, we believe that K will not be clarified by an approach such as cross-validation, which would still require manual interpretation. Overall, we feel that greater resolution (i.e. higher K) will allow for a more specific interpretation of the underlying biology. Nonetheless, if we set K = 2, we still observe enrichment (albeit less significant) for cilia biology among the meta-cluster composed of the overrepresented sgRNA (Cluster 1 and Cluster 2 from the K = 4 analysis). Thus, the conclusion that ciliary biology plays a role in the shared biology between ASD and CHD is not dependent on whether we choose four or two clusters, which represent the upper and lower limits of K that meet both the elbow plot and Silhouette method criteria.

- Normalization in Figure 3 should be explained clearly to be able to interpret the differences with Figure S4

We have elaborated on this normalization now in the methods (**LINES 378-382**): "We determined the percentage of ciliated cells in each image by dividing the number of cilia identified within a given image by the total number of cells present in the same image (**Fig. S4**). To normalize this measure, we divided the percent ciliated cells by the ratio of the number of cells in the image to the average BFP+ cell density of the non-targeting control sgRNA (**Fig. 3A, B, E**)."

- Costained images of hTAOKA-GFP cells, wildtype, and hTAOK1 transfected cells without GFP should be included in Figure S5. Otherwise it is unclear if overexpression of TAOK1 may lead to more fundamental changes in cilia formation

We now show co-stains and unmanipulated cells as controls. hTAOK1-GFP cells do not show cilia defects (**Fig. S6A-B**). We also now add a commercial TAOK1 antibody that shows similar localization, and validate it with our morpholino (**Fig. S6C-F**).

- Reporting participants from DECIPHER should be unbiased - how many total participants with de novo variants in TAOK1 were identified, and what proportion of them had CHD? Did any of them have other potential causal variants? Given that the Xenopus model had near complete deletion of Taok1, are you proposing a dominant negative mechanism for human disease?

In DECIPHER, we originally used the phenotype browser to identify phenotypes, including CNVs and other variants. Thanks to your comment, we have now more carefully analyzed these data. A total of 10 patients with pathogenic or likely pathogenic variants in TAOK1 are listed. 1 of these patients also has a likely pathogenic missense variant in *ZEB2*, which is known to cause Mowat-Wilson syndrome, which often presents with heart defects. Excluding this patient, of the 9 remaining patients, 1 has 'autistic behavior' listed, and many others have neurological conditions (including cilia-associated hydrocephalus). Several others have cardiac structural issues listed. However, we feel these data are limited given the nature of reporting and have instead just removed this sentence from the manuscript. We appreciate the reviewer bringing this to our attention.

Regarding gene dosage, we are not proposing a dominant negative mechanism. Rather, we assume a haploinsufficiency mechanism, common with ASD and CHD-associated de novo variants (Fu et al 2022, Jin et al 2017). As in many animal studies, it is far easier to identify robust phenotypes in strong loss of function than in haploinsufficiency, so for this work we have opted to take this approach to generate hypotheses to follow up in models where gene dosage can be more precisely considered (ie, germline mutants, etc).

<u>Second decision letter</u>

© 2025. Published by The Company of Biologists under the terms of the Creative Commons Attribution License (https://creativecommons.org/licenses/by/4.0/).

MS ID#: dev.204295R1

MS TITLE: Ciliary biology intersects autism and congenital heart disease

AUTHORS: Nia Teerikorpi, Kate E. McCluskey, Ethel Bader, Micaela C. Lasser, Sheng Wang, Catherine H. Nguyen, James D. Schmidt, Elina Kostyanovskaya, Nawei Sun, Jeanselle Dea, Tomasz J. Nowakowski, A. Jeremy Willsey and Helen Rankin Willsey

Dear Dr Willsey,

I have now received the reports on your revised manuscript of two of the referees who reviewed the initial version, and I have reached a decision. The referees' comments are appended below, or you can access them online: please go to .

The overall evaluation is positive and we would like to publish a revised manuscript in Development, provided that the referees' remaining comments and requests can be satisfactorily addressed. Please attend to all of these comments in your revised manuscript and detail them in your point-by-point response. If you do not agree with any of their criticisms or suggestions explain clearly why this is so.

Reviewer 1

Advance summary and potential significance to field

The revised manuscript addresses my major concerns by adding a CRISPR phenocopy of cilia phenotypes in Xenopus embryos resulting from TAOK1 disruption, consistent with the morpholino findings. The authors also show that TAOK1 is localized to cilia and show that the morpholino leads to reduced TAOK1 levels using immunohistochemistry. While I would have also liked for the authors to show phenocopy of the reduced brain and heart size phenotypes by CRISPR disruption of TAOK1, given that size phenotypes may be impacted by off-target effects of morpholinos, I think the authors provide sufficient evidence that the TAOK1 morpholino and CRISPR disruption phenocopy cilia phenotypes and that the morpholino leads to decreased TAOK1 levels in vivo. In addition, I appreciate the authors' adding a clarifying statement in the Discussion that their findings do not suggest that NPC proliferation is specific to the overlap of ASD and CHD genes and mainly show that ciliary biology might represent a point of convergence of genes associated with both conditions. I also appreciate the addition of cell cycle analyses showing that TAOK1 knockdown does not affect cell cycle progression, suggesting at least for this gene that the cilia phenotype is not due to alterations in the cell cycle. Overall, my impression is that the revised manuscript is improved and that the findings regarding the convergence of a subset of ASD and CHD on ciliary biology is impactful.

Comments for the author

Minor concerns—

1. It might be helpful to add to the Fig. 3 legend that the data were normalized. This was added to the main text but not to the figure legend.

2. It would be helpful to add to the Fig. 2 legend that the log2FC was based on > or = 0.585 for at least one timepoint. This is stated in the Fig. S1 legend but would be helpful to include here.

3. It is not clear if Fig. 4E and Fig. S7E are measuring the same area/intensity. Should the y axis labels be the same for these two graphs?

4. Table S3- It is not clear if this table provides all enrichments or only select enrichments for biological process/cellular components. If select, it would be helpful to also include all enrichments for these genes in addition to those related to ciliary biology.

© 2025. Published by The Company of Biologists under the terms of the Creative Commons Attribution License (https://creativecommons.org/licenses/by/4.0/).

5. Page 3, line 99- "Convergent" seems more accurate here than "congruent biological processes."

6. Fig. S6 - The sentence appears to be repeated: "(C - C'") Taok1 (green) antibody staining labels ciliary axonemes (Acetylated α-Tubulin, magenta) and the actin network (Phalloidin, gray) of the X. tropicalis epidermis. (C - C'") hTAOK1-GFP localizes to ciliary axonemes (Acetylated α-Tubulin, magenta) and the actin network (Phalloidin, gray) of the X. tropicalis epidermis." I think one of these statements refers to "B-B'"".

7. Page 8, lines 249-251- Both sentences start with "Nevertheless"; this is somewhat confusing.

Reviewer 3

Advance summary and potential significance to field

This manuscript identifies candidate CHD/ASD genes and highlighted their relevance to ciliary biology.

Comments for the author

All reviewer comments have been addressed and key experiments/analyses/edits completed which have improved the manuscript.

Second revision

Author response to reviewers' comments

We thank the editor and reviewers for the expedient review and all their work to improve the manuscript.

Comments from the Reviewers:

Reviewer 1: SUMMARY OF THE ADVANCE MADE IN THIS PAPER AND ITS POTENTIAL SIGNIFICANCE TO THE FIELD

The revised manuscript addresses my major concerns by adding a CRISPR phenocopy of cilia phenotypes in Xenopus embryos resulting from TAOK1 disruption, consistent with the morpholino findings. The authors also show that TAOK1 is localized to cilia and show that the morpholino leads to reduced TAOK1 levels using immunohistochemistry. While I would have also liked for the authors to show phenocopy of the reduced brain and heart size phenotypes by CRISPR disruption of TAOK1, given that size phenotypes may be impacted by off-target effects of morpholinos, I think the authors provide sufficient evidence that the TAOK1 morpholino and CRISPR disruption phenocopy cilia phenotypes and that the morpholino leads to decreased TAOK1 levels in vivo. In addition, I appreciate the authors' adding a clarifying statement in the Discussion that their findings do not suggest that NPC proliferation is specific to the overlap of ASD and CHD genes and mainly show that ciliary biology might represent a point of convergence of genes associated with both conditions. I also appreciate the addition of cell cycle analyses showing that TAOK1 knockdown does not affect cell cycle progression, suggesting at least for this gene that the cilia phenotype is not due to alterations in the cell cycle. Overall, my impression is that the revised manuscript is improved and that the findings regarding the convergence of a subset of ASD and CHD on ciliary biology is impactful.

We thank the reviewer for their thorough reading of the revised manuscript and for all their efforts in improving it.

SUGGESTIONS TO AUTHORS

© 2025. Published by The Company of Biologists under the terms of the Creative Commons Attribution License (https://creativecommons.org/licenses/by/4.0/).

Minor concerns—

1. It might be helpful to add to the Fig. 3 legend that the data were normalized. This was added to the main text but not to the figure legend.

This point is now included in the legend for Fig. 3, with an additional reference to Fig. S4 for the data before normalization.

2. It would be helpful to add to the Fig. 2 legend that the log2FC was based on > or = 0.585 for at least one timepoint. This is stated in the Fig. S1 legend but would be helpful to include here.

We have made this change.

3. It is not clear if Fig. 4E and Fig. S7E are measuring the same area/intensity. Should the y axis labels be the same for these two graphs?

For Fig. 4E, the intensity is normalized for the Centrin-positive area, since Centrin mRNA presence likely correlates with the area that has received the morpholino (since morpholinos usually travel similarly to mRNAs). However, in Fig. S7E, this is a CRISPR injection, and in our experience the Centrin-positive area does not always approximate the region that received mutagenesis (if anything, the Centrin-positive region is wider than the mutagenized region). Therefore we just quantified intensity per embryo. This likely underestimates the effect of the CRISPR, but nevertheless we still saw a significant change.

4. Table S3- It is not clear if this table provides all enrichments or only select enrichments for biological process/cellular components. If select, it would be helpful to also include all enrichments for these genes in addition to those related to ciliary biology.

This table provided all enrichments with FDR < 0.05. We have edited the legend to make this more clear.

5. Page 3, line 99- "Convergent" seems more accurate here than "congruent biological processes."

We have made this change.

6. Fig. S6 - The sentence appears to be repeated: "(C - C'") Taok1 (green) antibody staining labels ciliary axonemes (Acetylated α-Tubulin, magenta) and the actin network (Phalloidin, gray) of the X. tropicalis epidermis. (C - C'") hTAOK1-GFP localizes to ciliary axonemes (Acetylated α-Tubulin, magenta) and the actin network (Phalloidin, gray) of the X. tropicalis epidermis." I think one of these statements refers to "B-B'"".

We have fixed this legend by deleting the second sentence, since it does indeed refer to B, which was already described.

7. Page 8, lines 249-251- Both sentences start with "Nevertheless"; this is somewhat confusing.

We have deleted the second "nevertheless."

Reviewer 3: SUMMARY OF THE ADVANCE MADE IN THIS PAPER AND ITS POTENTIAL SIGNIFICANCE TO THE FIELD

This manuscript identifies candidate CHD/ASD genes and highlighted their relevance to ciliary biology.

SUGGESTIONS TO AUTHORS

All reviewer comments have been addressed and key experiments/analyses/edits completed which have improved the manuscript.
We thank the reviewer for their time and energy in improving this manuscript.

© 2025. Published by The Company of Biologists under the terms of the Creative Commons Attribution License (https://creativecommons.org/licenses/by/4.0/).

Third decision letter

MS ID#: dev.204295R2

MS TITLE: Ciliary biology intersects autism and congenital heart disease

AUTHORS: Nia Teerikorpi, Kate E. McCluskey, Ethel Bader, Micaela C. Lasser, Sheng Wang, Catherine H. Nguyen, James D. Schmidt, Elina Kostyanovskaya, Nawei Sun, Jeanselle Dea, Tomasz J. Nowakowski, A. Jeremy Willsey and Helen Rankin Willsey

Dear Dr Willsey,

I am delighted to tell you that your manuscript has been accepted for publication in Development, pending our standard publication integrity checks.

© 2025. Published by The Company of Biologists under the terms of the Creative Commons Attribution License (https://creativecommons.org/licenses/by/4.0/).
